# Sporopollenin-inspired design and synthesis of robust polymeric materials

**DOI:** 10.1038/s42004-022-00729-w

**Published:** 2022-09-12

**Authors:** Christopher M. Glinkerman, Shaoting Lin, Jiahua Ni, Fu-Shuang Li, Xuanhe Zhao, Jing-Ke Weng

**Affiliations:** 1 Whitehead Institute for Biomedical Research, Cambridge, MA 02142 USA; 2 Department of Mechanical Engineering, Massachusetts Institute of Technology, Cambridge, MA 02139 USA; 3 Department of Biology, Massachusetts Institute of Technology, Cambridge, MA 02139 USA

**Keywords:** Polymer synthesis, Bioinspired materials

## Abstract

Sporopollenin is a mechanically robust and chemically inert biopolymer that constitutes the outer protective exine layer of plant spores and pollen grains. Recent investigation of the molecular structure of pine sporopollenin revealed unique monomeric units and inter-unit linkages distinct from other previously known biopolymers, which could be harnessed for new material design. Herein, we report the bioinspired synthesis of a series of sporopollenin analogues. This exercise confirms large portions of our previously proposed pine sporopollenin structural model, while the measured chemical, thermal, and mechanical properties of the synthetic sporopollenins constitute favorable attributes of a new kind of robust material. This study explores a new design framework of robust materials inspired by natural sporopollenins, and provides insights and reagents for future elucidation and engineering of sporopollenin biosynthesis in plants.

## Abstract (web summary)

Pine sporopollenin harbors unique monomeric units and inter-unit linkages forming mechanically robust and chemically inert biopolymers. Here, the authors design and synthesize several sporopollenin analogues, and characterize their chemical, thermal and mechanical properties as robust polymers.

## Introduction

**Fig. 1:**
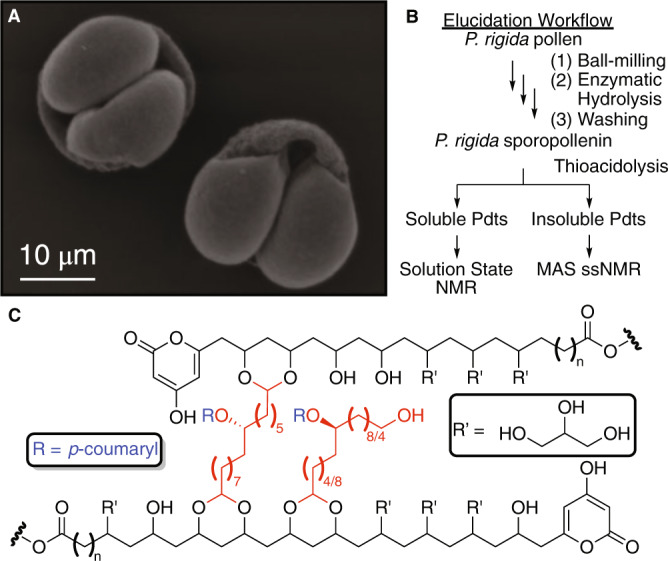
Summary of the current structural model of pine sporopollenin. **A** Electron micrograph of *P. rigida* pollen^35^, **B** workflow employed in the structural elucidation of *P. rigida* sporopollenin, and **C** previously proposed ^13^C MAS ssNMR averaged structure of *P. rigida* sporopollenin (structural notes: pyrone may be substituted by an ester moiety, approximately 15% of aliphatic units are singly crosslinked, higher dimensional crosslinking is likely, the linkage between R’ and the backbone is currently unknown and has been purposefully left ambiguous herein)^35^.

Sporopollenin (the portmanteau^1,2^ of the archaic “sporonin^3^” and “pollenin^4^”) is the general designation for a class of chemically related and ubiquitous biopolymers that comprise the exine of plant spores and pollen grains (Fig. 1A)^1–8^. These exines adopt a wide variety of complex supramolecular structures ranging widely in size, shape, and porosity^1–8^. In its natural role, sporopollenin serves to protect the fragile gametes of land plants against myriad environmental insults including desiccation, ultraviolet irradiation, chemical degradation, and mechanical stress^5–8^. As a result, sporopollenin has evolved to be one of the strongest and most chemically resistant known materials of direct biological origin boasting elastic moduli as high as 16 ± 2.5 GPa^9^, resistance to hydrostatic pressures in excess of 10 GPa^10^, and inertness towards a wide variety of organic solvents^5–8,11^. Due to the inexorable nature of the supramolecular and molecular structure of natural sporopollenins, it is difficult to deconvolute the exact contributions of each to the aforementioned properties. Nevertheless, these properties have led to the successful application of natural sporopollenin to chromatography^12–14^, solid phase peptide synthesis^15^, catalyst solid supports^16–18^, magnetic nanoparticle synthesis^19,20^, and the encapsulation of enzymes^21,22^, pharmaceuticals^23–26^, and whole cells^27^. Despite these early successes, the implementation of sporopollenin-based technologies has been stymied by an historically limited understanding of the molecular structure of sporopollenin and lack of consistent supply of nature-derived sporopollenin materials.

For over 200 years, the chemical community has painstakingly engaged in structural elucidation studies of plant sporopollenins spanning myriad degradative, spectroscopic, and spectrometric methodologies with limited progress^1–8,11,28–35^. Recently, our own efforts in this area have culminated in the most detailed hypothesis for the molecular structure of *P. rigida* sporopollenin to date, facilitated by the implementation of ^13^C magic angle spinning solid state nuclear magnetic resonance (MAS ssNMR) spectroscopy and degradative thioacidolysis/liquid chromatography-mass spectrometry (DT/LC-MS) (Fig. 1B)^35^. These studies suggest that *P. rigida* sporopollenin is principally comprised of aliphatic polyketide-derived polyvinyl alcohol units crosslinked by *p*-coumaryl-substituted fatty acid-derived C16 aliphatic units via acetal linkages (Fig. 1C)^35^. This proposed structure has withstood recent spectroscopic scrutiny^28^ and, therefore, represents a validated starting point for synthesis of structural analogues that retain sporopollenin’s fascinating properties.

Herein, we describe a synthetic strategy towards the preparation of a library of bioinspired synthetic sporopollenin analogues and the chemical, thermal, and mechanical assessment thereof. As a result of this work, we have (1) validated significant portions of our recently proposed^35^ molecular structure of *P. rigida* sporopollenin through chemical synthesis, (2) provided general access to an underexplored class of bioinspired and biocompatible polymers, (3) identified key structure-property relationships (SPRs) enabling purpose-guided design of bespoke sporopollenin analogues, and (4) established chemical tools for the elucidation of sporopollenin biosynthesis empowering future development of ectopic sporopollenin-accumulating organisms for carbon sequestration. Through these efforts, we aim to further demystify one of Nature’s most robust materials and facilitate its potential commercial application in the areas of pharmaceutical encapsulation, anti-fouling agents, and chemically-inert coatings, *inter alia*.

## Results and discussion

### Chemical synthesis of sporopollenin-inspired polymers

Thus, a library of bioinspired synthetic sporopollenin analogues was envisioned to arise from the well-precedented acid-catalyzed crosslinking of commercial polyvinyl alcohol^36–42^ with a suite of synthetically accessed α,ω-dialdehydes^43^. The central feature of this approach is the divergent preparation of variously substituted α,ω-dialdehyde crosslinkers via either oxidation of simple α,ω-alkanols or an alkylation/ring expansion sequence that permits variability of chain length, substituent identity/position, and chirality. This approach would permit the rapid and divergent preparation of myriad sporopollenin-like polymers with discrete control over linker length, linker substituent(s), degree of crosslinking, and polyvinyl alcohol backbone properties, including average molecular weight, polydispersity, and tacticity.

**Fig. 2:**
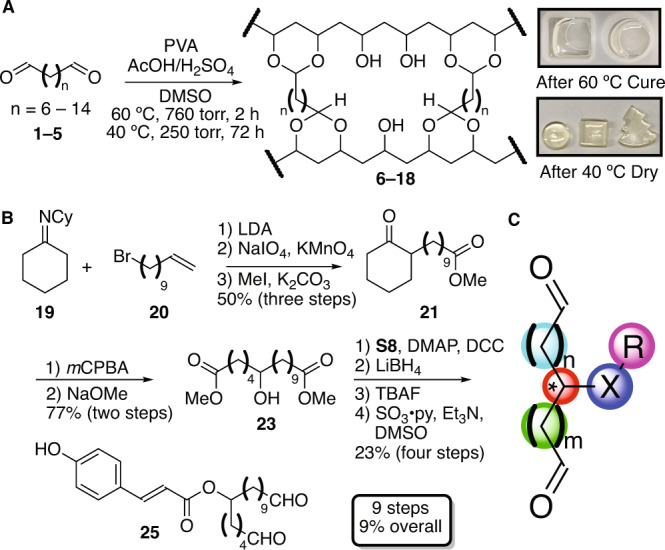
Chemical synthesis of sporopollenin analogues. **A** Simplified sporopollenin analogue **6**–**18** synthesis, **B** synthetic preparation of substituted crosslinker **25**, and **C** summary of potential crosslinker analogue route divergence (teal/green: linker length modifiable via selection of cyclic ketone and bromoalkene, red: stereochemistry set via enantioselective alkylation or ring expansion, blue: heteroatom altered via selection of ring expansion conditions, purple: substituent varied via esterifications, amide couplings, alkylations, *inter alia*).

To those ends, a series of unsubstituted α,ω-dialdehydes (**1**–**5**), prepared by oxidation of the corresponding commercially available α,ω-alkanols (**S1**–**S5**)^43^, were crosslinked with 5% polyvinyl alcohol (PVA) in DMSO under acid-catalyzed conditions (Fig. 2A)^36–42^. While a temperature of ≥55 °C was required for efficient crosslinking, extended periods of drying at ≥55 °C resulted in pronounced discoloration of the crosslinked material. This is likely due to the known acid-catalyzed decomposition of DMSO at elevated temperature^44,45^. In order to minimize the impact of this undesired side-reaction, polymers were cured at 55–60 °C and 760 torr for 2 h under air, then dried at 40 °C and 250 torr for 72 h under air resulting in nearly colorless, transparent crosslinked polymers. With an efficient synthetic methodology in hand, a suite of simplified sporopollenin analogues with theoretical degrees of crosslinking ranging from 5–50% (Table S1), crosslinker length ranging from C8-C16, and PVA average molecular weight ranging from 31,000–186,000 g/mol were prepared for physical, thermal, and chemical evaluation, totaling 13 discrete analogues (**6**–**18**, see Supplementary Methods, Supplementary Note 1, and Table S2 for numbering convention).

**Fig. 3:**
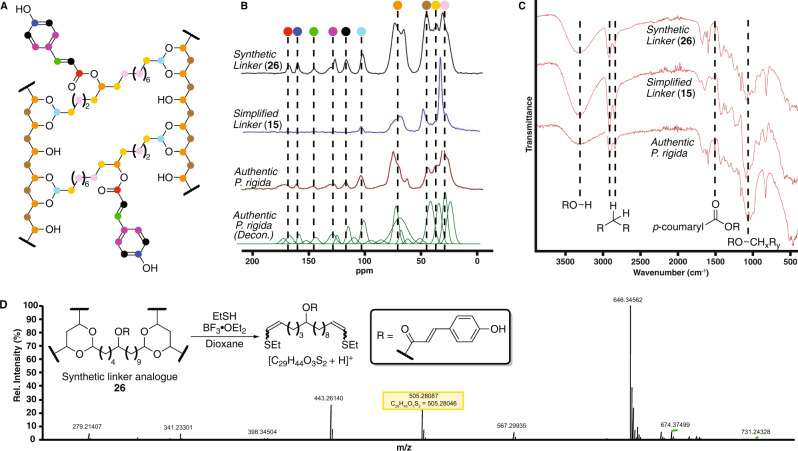
Structural analyses of synthetic sporopollenin analogues. **A** Molecular structure of synthetic sporopollenin analogue **26**, **B** ^13^C MAS ssNMR comparison (black: synthetic linker **26** analogue, blue: simplified linker **15** analogue, red: authentic *P. rigida* sporopollenin, green: computationally deconvoluted authentic *P. rigida* sporopollenin)^35^, **C** ATR-FTIR comparison (top: synthetic linker **26** analogue, middle: simplified linker **15** analogue, bottom: authentic *P. rigida* sporopollenin), and **D** DT/LC-HRMS analysis of synthetic sporopollenin **26**.

While these simplified sporopollenin analogues facilitated the optimization of the aforementioned curing method and the rapid establishment of preliminary structure-property relationships, we sought to more closely reproduce the authentic sporopollenin linker to both test the validity of the prior *P. rigida* sporopollenin structural model^35^ and provide precedent for the divergent preparation of myriad functionalized linkers bearing substituents both natural and previously unobserved in sporopollenin across plant families (Fig. 2B)^1–8,11,28–35^. Accordingly, alkylation of cyclohexylimine **19**^46^ with bromoolefin **20** (^*n*^BuLi, DIPA, THF, 0–23 °C, 22 h, 69%) provided, after subsequent oxidative cleavage (NaIO_4_, KMnO_4_, acetone/H_2_O, 23 °C, 20 h, 81%) and alkylation (MeI, K_2_CO_3_, acetone, reflux, 24 h, 89%), ester **21**. Ring expansion under Baeyer-Villiger conditions^47,48^ afforded the corresponding caprolactone **22** (*m*CPBA, CH_2_Cl_2_, 0–23 °C, 20 h, 91%) which underwent smooth one-pot ring opening and esterification to afford diester **23** (NaOMe, MeOH, 23 °C, 3.5 h, 85%). This simultaneously exposed a key alcohol moiety for further functionalization. Steglich esterification^49^ with TBS-protected *p*-coumaric acid (**S8**)^50,51^ provided triester **24** (**S8**, DMAP, DCC, CH_2_Cl_2_, 23–40 °C, 20 h, 69%), which, after reduction (LiBH_4_, Et_2_O, 23 °C, 3 h, 64%), deprotection (TBAF, THF, 23 °C, 5 min, 90%), and oxidation (SO_3_·py, Et_3_N, DMSO/CH_2_Cl_2_, 0 °C, 2.5 h, 58%), was converted to the target dialdehyde **25** (9 steps from commercial, 9% overall yield, see Figs. S1–S10 for spectra). It is notable that many intermediates en route to dialdehyde **25** (e.g., **23**, **24**) are closely related to proposed biosynthetic pathway intermediates and, thus, may be implemented in the future validation of sporopollenin biosynthetic hypotheses^52–61^. While yet unexplored, we anticipate this synthetic route will permit rapid divergence to myriad sporopollenin linker analogues in the future (Fig. 2C). Analogous to the simple α,ω-dialdehyde crosslinkers^43^, synthetically accessed dialdehyde **25** was crosslinked with PVA^36–42^ to afford synthetic linker sporopollenin analogue **26** (Fig. 3A), representing the closest synthetic recapitulation of *P. rigida* sporopollenin disclosed to date^35^.

### Spectroscopic characterization and chemical stability of sporopollenin-inspired polymers

Initial comparison of synthetic analogues **15** and **26** with authentic *P. rigida* sporopollenin^35^ revealed a high degree of similarity in both ^13^C MAS ssNMR^29,62^ (Fig. 3B) and attenuated total reflectance Fourier-transform infrared (ATR-FTIR, Fig. 3C) spectroscopies (Figs. S11–18)^28,63,64^. The presence of acetal cross-linkages in all three samples was confirmed by a broad NMR signal from 97–103 ppm corroborated by an IR band at ~1100 cm^−1^ corresponding to an asymmetric stretching of aliphatic ethers. The broadness of both signals coupled with the fine structure of the IR band suggest the acetals are present in a variety of subtly distinct chemical environments as anticipated in a highly crosslinked, three-dimensional polymeric structure. Further mutual upfield NMR signals from 62–80 ppm and 20–52 ppm were consistent with oxygen-bearing and aliphatic methylene carbons, respectively, attributed to the skipped polyol backbone and aliphatic crosslinkers. The latter is further corroborated by the presence of methylene C–H asymmetric and symmetric stretches in the IR spectra at 2920 and 2850 cm^−1^, respectively. More detailed comparison of synthetic linker sporopollenin analogue **26** and authentic *P. rigida* sporopollenin revealed a series of remarkably similar downfield NMR signals corresponding to the arene (111–121, 123–135, 155–162 ppm), olefin (111–121, 164–170 ppm), and carbonyl (164–170 ppm) of the *p*-coumaryl ester moiety with a sharp IR signal at ~1514 cm^−1^ attributed to an aromatic ring mode of the coumaryl ester^28^. As chemical moieties previously predicted to be secondary to the structural linkages of sporopollenin were not included in current synthetic efforts, NMR signals at 87 and 96 ppm, diagnostic of α-pyrones, were observed only in the authentic *P. rigida* sporopollenin^35^. Curiously, a medium to sharp IR signal at ~830 cm^−1^ previously attributed in *P. ponderosa* sporopollenin to an aromatic CH out-of-plane bending^28^ is present in all three samples, suggesting a nonaromatic origin. In addition to the highlighted signals, the IR fingerprint regions across samples demonstrate excellent overlap strongly supporting the highly similar nature of the authentic and synthetic samples.

In analogy to our prior *P. rigida* sporopollenin structural elucidation efforts, synthetic analogues **15** and **26** were submitted to degradative thioacidolysis followed by liquid chromatography-high resolution mass spectrometry (DT/LC-HRMS) analysis^35^, and compared with authentic material to assess similarities in chemical reactivity (Fig. 3D, Figs. S19–20). Spectrometry revealed the presence of analogous bis-thioenol ether degradation products in each sample, likely resulting from the in-source fragmentation of the corresponding bis-dithioacetal of the relevant crosslinking α,ω-dialdehyde, suggesting similar mechanisms of degradation across synthetic (**15**, **26**) and authentic sporopollenins^35^. The chemical stability of analogue **15** was further examined by calculating mass differences in samples exposed to organic solvents over 24 h. These solvolysis studies revealed no significant mass changes suggesting broad resistance of sporopollenin analogues to organic solvents, consistent with prior reports of the chemical resilience of natural sporopollenins (see Table S3)^11^.

Taken together, the spectroscopic, spectrometric, and chemical reactivity data across synthetic sporopollenin analogues **15** and **26** bear remarkable similarities to authentic *P. rigida* sporopollenin^35^, thus demonstrating, through chemical synthesis, the accuracy of large portions of our previous structural hypothesis, and providing access to both an underexplored class of bioinspired and biocompatible polymer and tools for sporopollenin biosynthetic pathway elucidation^52–61^. While independent ^13^C MAS ssNMR studies are currently unreported, the ATR-FTIR data disclosed herein are broadly consistent with recent analyses of *P. ponderosa* sporopollenin by ref. ^28^ and suggest high structural similarity between *P. rigida* and *P. ponderosa* sporopollenin.

### Thermal and mechanical characterization of sporopollenin-inspired polymers

With our structural hypothesis for *P. rigida* sporopollenin largely confirmed, we set out to examine the thermal and mechanical properties of the synthetic sporopollenin analogues. Prior studies on a variety of natural sporopollenins have revealed appealing properties including high modulus^9^ and resistance to significant hydrostatic pressures^10^. Though direct comparison of natural and synthetic sporopollenins is not yet possible due to differences in supramolecular structure, we anticipated synthetic analogues would exhibit broadly similar properties to their natural counterparts that would permit wide application in materials science. To those ends, thermogravimetric analysis (TGA) was conducted on all analogues disclosed to ascertain their thermal stability and extent of contamination by low molecular weight solvents and process aids (Table S9, Figs. S44–S57). Across all analogues, a series of three mass losses of 34.6 ± 2.4% at 213 ± 9.8 °C, 54.1 ± 4.0% at 453 ± 5.9 °C, and 9.8 ± 1.4% at 535 ± 8.0 °C corresponded to loss of residual solvents and process aids, thermal decomposition of the base polymer, and combustion, respectively. Differential scanning calorimetry (DSC) was also conducted on simplified sporopollenin analogue **15**, which revealed no significant thermal features below 110 °C, above which data interpretation was complicated by the loss of residual solvent (Figs. S58–S62). Taken together, these data demonstrate that the method for polymer crosslinking is consistent in producing sporopollenin analogues containing approximately 35 wt/wt% DMSO which resist thermal decomposition to approximately 453 °C and do not exhibit supramolecular organization into crystalline or semi-crystalline domains.

**Fig. 4:**
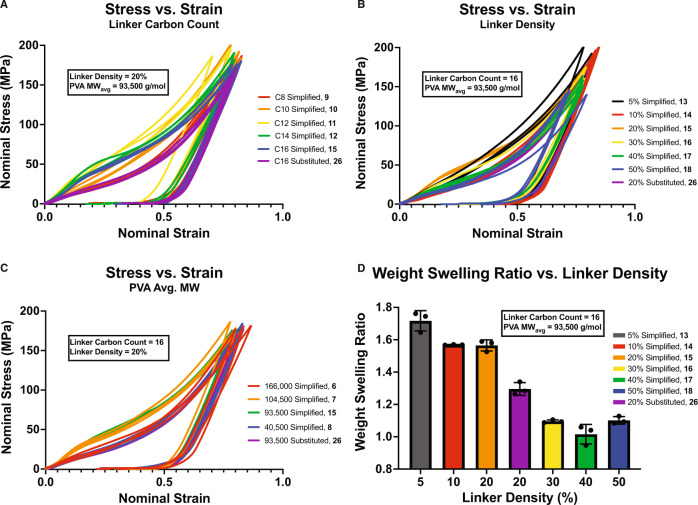
Comparison of representative compressive stress-strain curves of synthetic sporopollenin analogues. **A** crosslinker length, **B** crosslinker density, and **C** PVA average molecular weight and **D** relationship between weight swelling ratio and crosslinker density (*n* = 3, error bars = standard deviation).

In addition to thermal analysis, all synthetic sporopollenin analogues disclosed were submitted to a battery of mechanical property evaluations to assess both similarity to previously disclosed properties of natural sporopollenins^9^ and potential commercial utility as structurally sound polymeric materials. Durometer hardness testing of the analogues revealed almost uniform results averaging 71 ± 5 Shore D regardless of crosslinker length, crosslinking density, and PVA average molecular weight (Table S4). This value is higher than that of high-density polyethylene (65 Shore D)^65^, commonly utilized in commercial thermoplastic hard hats. Compression testing of the analogues was performed with controlled crosslinker length, crosslinker density, and PVA average molecular weight up to a stress of approximately 200 MPa, measuring their nominal stress versus nominal strain curves, Young’s moduli, and hysteresis ratios (Tables S5–S6, Figs. S21–S40). Compressive moduli ranged from 97 ± 8 MPa to 230 ± 36 MPa across all analogues with a crosslinker length of C12 yielding the maximal observed modulus value (Fig. 4A). Due to the low solvent content of the polymers, both elastically active chain density and inter-/intramolecular interactions likely contribute to the moduli of all analogues^66^. Since crosslinker density is proportional to elastically active chain density but inversely proportional to inter-/intramolecular interactions, there is no strong dependence of moduli on crosslinker density observed experimentally (Fig. 4B). In addition, no strong dependence on PVA average molecular weight on moduli was observed (Fig. 4C), which suggests inter-/intramolecular interactions of analogues with different PVA average molecular weights are similar. It is notable that in all instances observed, synthetic analogue **26** demonstrated decreased modulus compared to the analogous simplified synthetic analogue **15**, suggesting that the presence of *p*-coumaryl substituents suppresses inter-/intramolecular interactions of analogues, thereby leading to reduced modulus^67^. The hysteresis ratio across analogues varied between 0.48 ± 0.01 and 0.63 ± 0.01, indicating significant dissipation of energy over one compressive cycle. The dissipation of energy is recoverable (Fig. S42). In addition, large plasticity and rate dependency are observed across all analogues in tensile testing (Figs. S41, S43). The recoverable dissipation of energy, large plasticity, and rate dependency suggests the presence of residual acetic or sulfuric acid, utilized as a catalyst for polymer crosslinking, renders the dynamic forming and reforming of crosslinks between α,ω-dialdehydes and PVA chains at highly deformed states. In addition to compression and tensile tests, the swelling ratios of all analogues were also measured (Tables S7–S8). As shown in Fig. 4D, the weight swelling ratio decreases from 1.71 to 1.02 as crosslinker density increases from 5 to 50%. Notably, a plateau in the decrease in swelling ratio with increasing crosslinker density occurs between 20 and 30% crosslinking; the same level of crosslinking previously observed in natural *P. rigida* sporopollenin^35^. This suggests that plants might have been subject to evolutionary pressure to minimize the swelling of their sporopollenin and demonstrates that they have successfully adapted in the most efficient manner possible. No clear relationship between swelling ratio and PVA average molecular weight or crosslinker length were observed. Overall, these thermal and mechanical analyses reveal the unique properties of natural and unnatural sporopollenin analogues and their potential to broadly impact the field of materials science.

### Broader impact, limitations, and future directions

Plant molecular biologists have long searched for plant genes likely involved in plant sporopollenin biosynthesis^52–61^. At present, at least 11 enzyme-encoding genes from the model plant *Arabidopsis thaliana* have been implicated, on the basis of single and multiple mutant phenotypes, in pollen exine development^52–61^. These genes include fatty acid reductase *MALE STERILITY 2 (MS2)*^53^, *ACYL-COA SYNTHETASE 5 (ACOS5)*^54^, *POLYKETIDE SYNTHASE A/B (PKSA/B)*^55,56^, *TETRAKETIDE ɑ-PYRONE REDUCTASE 1/2 (TKPR1/2)*^57,58^, *LESS ADHESIVE POLLEN 3 (LAP3)*^59^, *CYP703A2*^60^, *CYP704B1*^61^, and *IRREGULAR POLLEN EXINE 1/2 (IPE1/2)*^52^. As the precise roles and ordering of these enzymes in the sporopollenin biosynthetic pathway remain an open field for future research^52–61^, we note that our current study provides a framework for synthesizing a variety of isotopically labeled, stable pathway intermediates that can be used as chemical tools to probe various sporopollenin biosynthetic hypotheses. Such efforts may ultimately enable bioengineering of sporopollenin biosynthesis in non-reproductive tissues of plants with potential applications ranging from enhancing plant biotic and abiotic stress resistance to carbon sequestration^52–61^.

Though the work herein provides detailed evaluation of numerous synthetic sporopollenin analogues, it is noteworthy that direct comparison of some properties of natural and synthetic sporopollenins remains out of reach due, in large part, to the intricate supramolecular structure of the natural material (Fig. 1A)^1–8,35^. For example, compressive testing of natural sporopollenin provides moduli that likely reflect contributions from both molecular and supramolecular structural elements and, thus, cannot be directly compared to a result from the synthetic samples which, while comparable in molecular structure, lack a similar supramolecular shape. Only evaluation of the properties of the powdered samples are directly comparable as the supramolecular structures are disrupted. To achieve a more direct comparison, it will be necessary to achieve discrete control over supramolecular structure during the synthesis of sporopollenin analogues. Studies towards these ends are ongoing. In addition, future efforts may include exploration of alternative crosslinker substituents with the goal of imparting the polymers with auxiliary properties (i.e., larger π-systems for optical properties).

In summary, we report the first synthetic recapitulation of plant sporopollenin and analogues thereof based upon that of *P. rigida*^35^. The synthetic sporopollenin analogues harbor numerous favorable chemical, thermal, and mechanical properties of robust polymers with potential industrial applications. These efforts have resulted in the validation of significant portions of our previous structural model for *P. rigida* sporopollenin^35^, granted chemical access to an underexplored class of bioinspired and biocompatible polymers, revealed key structure-property relationships for the engineering of sporopollenin analogues, and provided chemical tools for the future elucidation of sporopollenin biosynthesis^52–61^. This work affords foundational principles which will inform the development and application of robust sporopollenin-inspired polymers in the areas of pharmaceutical encapsulation, anti-fouling agents, and chemically-inert coatings while simultaneously demystifying one of Nature’s most enigmatic materials.

## Methods

### General method for the preparation of α,ω-Alkyldialdehydes (1–5)

A solution of α,ω-alkyldiol **S1–S5** (1.00 mmol) in CH_2_Cl_2_ (10.0 mL) at 23 °C was treated with DMP (2.20 mmol). The resulting solution was stirred at 23 °C for 2 h, then diluted to a total volume of 110 mL with Et_2_O, washed sequentially with 1 N NaOH(aq) (2 × 100 mL) and sat. NH_4_Cl(aq) (1 × 100 mL), dried over Na_2_SO_4_, concentrated on a rotary evaporator, and purified by flash chromatography (SiO_2_, 20–50% Et_2_O/Hexanes) to provide α,ω-alkyldialdehydes (**1**–**5**) as clear, colorless oils (**5**) or amorphous white solids (**1**–**4**)^43^.

**1,16-Hexadecanedial (1)**^68^. ^1^H NMR (CDCl_3_, 400 MHz) δ 9.76 (t, *J* = 1.9 Hz, 2H), 2.41 (td, *J* = 7.4, 1.9, 4H), 1.65–1.58 (m, 4H), 1.38–1.22 (m, 20H).

**1,14-Tetradecanedial (2)**^69^. ^1^H NMR (CDCl_3_, 500 MHz) δ 9.76 (t, *J* = 1.9 Hz, 2H), 2.42 (td, *J* = 7.4, 1.9 Hz, 4H), 1.63 (p, *J* = 7.4 Hz, 4H), 1.36–1.23 (m, H16).

**1,12-Dodecanedial (3)**^70^. ^1^H NMR (CDCl_3_, 500 MHz) δ 9.77 (s, 2H), 2.42 (td, *J* = 7.6, 1.9 Hz, 4H), 1.63 (p, *J* = 7.4 Hz, 4H), 1.41–1.23 (m, 12 H).

**1,10-Decanedial (4)**^71^. ^1^H NMR (CDCl_3_, 500 MHz) δ 9.76 (s, 2H), 2.44 (dt, *J* = 7.3, 1.8 Hz, 4H), 1.65–1.60 (m, 4H), 1.40–1.22 (m, 8H).

**1,8-Ocatanedial (5)**^72^. ^1^H NMR (CDCl_3_, 500 MHz) δ 9.77 (t, *J* = 1.5 Hz, 2H), 2.44 (td, *J* = 7.3, 1.5 Hz, 4H), 1.64 (t, *J* = 7.3, 4H), 1.40–1.33 (m, 4H).

### 2-(Undec-10-en-1-yl)cyclohexan-1-one (S6)

A solution of freshly distilled diisopropylamine (6.77 mL, 48.0 mmol) in dry THF (575 mL) at 0 °C was treated dropwise with 2.45 M ^*n*^BuLi in hexanes (16.3 mL, 40.0 mmol) over 5 min. The resulting solution was stirred at 0 °C for 10 min and then treated with a solution of freshly distilled *N*-cyclohexylcyclohexanimine (**19**)^46^ (5.75 g, 32.1 mmol) in dry THF (50.0 mL). The bright yellow solution, thus obtained, was stirred at 0 °C for 1 h and then treated with a solution of 11-bromoundec-1-ene (**20**) (3.73 g, 16.0 mmol) in dry THF (100 mL). The reaction mixture was warmed to 23 °C and allowed to stir at 23 °C for 20 h. After 20 h, the resulting solution was poured into sat. NH_4_Cl(aq) (300 mL) and extracted with EtOAc (3 × 200 mL). The combined organic layers were washed with 1 N HCl(aq) (1 × 200 mL) and sat. NaCl(aq) (200 mL), dried over Na_2_SO_4_, concentrated on a rotary evaporator, and purified by flash chromatography (SiO_2_, 5% Et_2_O/Pet. Ether) to provide alkene **S6** as a pale yellow oil (2.75 g, 69%): ^1^H NMR (CDCl_3_, 400 MHz) δ 5.81 (ddt, *J* = 17.0, 10.2, 6.7 Hz, 1H), 4.99 (dq, *J* = 17.2, 1.7 Hz, 1H), 4.93 (ddt, *J* = 10.2, 2.4, 1.3 Hz, 1H), 2.38 (dtd, *J* = 13.6, 4.3, 1.3 Hz, 1H), 2.32–2.22 (m, 2H), 2.13–2.07 (m, 1H), 2.06–1.99 (m, 3H), 1.87–1.82 (m, 1H), 1.80–1.72 (m, 1H), 1.71–1.60 (m, 2H), 1.42–1.33 (m, 3H), 1.29–1.16 (m, 13H); ^13^C NMR (CDCl_3_, 100 MHz) δ 213.8, 139.4, 114.2, 50.9, 42.1, 34.0, 29.9, 29.7, 29.7, 29.6, 29.6, 29.3, 29.1, 28.2, 27.3, 25.0; IR (film) ν_max_ 2923, 2853, 1710, 1640, 1462, 1448, 1126, 993, 908 cm^−1^; HRMS (DART-TOF) *m/z* 251.2390 (C_17_H_30_O + H^+^ requires 251.2375).

### 10-(2-Oxocyclohexyl)decanoic acid (S7)

A solution of KMnO_4_ (42.6 mg, 0.270 mmol) in 1:6 acetone/H_2_O (13.5 mL) at 23 °C was treated with NaIO_4_ (2.31 g, 10.8 mmol), then dropwise with neat alkene **S6** (135 mg, 0.539 mmol) over 5 min. The resulting solution was stirred at 23 °C for 20 h, then diluted to 200 mL total volume with H_2_O and extracted with EtOAc (2 × 100 mL). The combined organic layers were dried over Na_2_SO_4_, concentrated on a rotary evaporator, and purified by flash chromatography (SiO_2_, 35% Et_2_O/Pet. Ether) to provide carboxylic acid **S7** as an amorphous white solid (109 mg, 81%): mp 39–42 °C; ^1^H NMR (CDCl_3_, 400 MHz) δ 10.57 (bs, 1H), 2.41–2.22 (m, 5H), 2.12–2.00 (m, 2H), 1.87–1.59 (m, 6H), 1.43–1.15 (m, 14H); ^13^C NMR (CDCl_3_, 100 MHz) δ 214.0, 180.0, 50.9, 42.1, 34.2, 34.0, 29.8, 29.6, 29.5, 29.5, 29.3, 29.2, 28.2, 27.3, 24.9, 24.8; IR (film) ν_max_ 2917, 2849, 1700, 1429, 1289, 1216, 952 cm^−1^; HRMS (DART-TOF) *m/z* 269.2137 (C_16_H_28_O_3_ + H^+^ requires 269.2117).

### Methyl 10-(2-Oxocyclohexyl)decanoate (21)

A solution of carboxylic acid **S7** (372 mg, 1.39 mmol) in acetone (14.2 mL) at 23 °C was treated sequentially with MeI (430 μL, 6.93 mmol) and K_2_CO_3_ (958 mg, 6.93 mmol). The resulting solution was refluxed for 24 h, then diluted with EtOAc to a total volume of 50 mL, washed with 1 N HCl(aq) (1 × 50 mL), dried over Na_2_SO_4_ and concentrated on a rotary evaporator. The resulting residue was purified by flash chromatography (SiO_2_, 20% Et_2_O/Pet. Ether) to provide methyl ester **21** as a pale yellow oil (349 mg, 89%): ^1^H NMR (CDCl_3_, 400 MHz) δ 3.66 (s, 3H), 2.38 (dt, *J* = 14.0, 4.5 Hz, 1H), 2.32–2.23 (m, 4H), 2.13–1.97 (m, 2H), 1.87–1.57 (m, 6H), 1.44–1.14 (m, 14H); ^13^C NMR (CDCl_3_, 100 MHz) δ 213.8, 174.5, 51.6, 50.9, 42.1, 34.3, 34.0, 29.9, 29.6, 29.5, 29.5, 29.4, 29.3, 28.2, 27.3, 25.1, 25.0; IR (film) ν_max_ 2925, 2854, 1737, 1709, 1448, 1435, 1196, 1170 cm^−1^; HRMS (DART-TOF) *m/z* 283.2309 (C_17_H_30_O_3_ + H^+^ requires 283.2273).

### Methyl 10-(7-Oxooxepan-2-yl)decanoate (22)

A solution of methyl ester **21** (78.6 mg, 0.278 mmol) in CH_2_Cl_2_ (1.40 mL) at 0 °C was treated with *m*CPBA (125 mg, 0.556 mmol). The resulting solution was warmed to 23 °C and stirred at 23 °C for 20 h. After 20 h, the reaction mixture was diluted with CH_2_Cl_2_ to a total volume of 3 mL, washed with sat. NaHCO_3_(aq) (2 × 3 mL), dried over Na_2_SO_4_, concentrated on a rotary evaporator, and purified by flash chromatography (SiO_2_, 20–40% Et_2_O/Hexanes) to provide caprolactone **22** as an amorphous white solid (75.3 mg, 91%): mp 29–31 °C; ^1^H NMR (CDCl_3_, 400 MHz) δ 4.21 (dt, *J* = 8.2, 3.9 Hz, 1H), 3.65 (s, 3H), 2.62 (qd, *J* = 13.7, 8.7 Hz, 2H), 2.29 (t, *J* = 7.6 Hz, 2H), 1.94–1.86 (m, 3H), 1.74–1.41 (m, 8H), 1.34–1.25 (m, 11H); ^13^C NMR (CDCl_3_, 100 MHz) δ 175.9, 174,4, 80.7, 51.6, 36.5, 35.1, 34.7, 34.2, 29.5, 29.5, 29.4, 29.3, 29.2. 28.5, 25.5, 25.1, 23.2; IR (film) ν_max_ 2925, 2855, 1726, 1437, 1172, 1011 cm^−1^; HRMS (DART-TOF) *m/z* 299.2270 (C_17_H_30_O_4_ + H^+^ requires 299.2222).

### Dimethyl 6-Hydroxyhexadecanedioate (23)

To neat caprolactone **22** (1.27 g, 4.26 mmol) at 23 °C was added a 0.5 M solution of NaOMe in MeOH (42.0 mL, 21.0 mmol). The resulting solution was stirred at 23 °C for 3.5 h. After 3.5 h, the reaction mixture was poured into sat. NH_4_Cl(aq) (100 mL) and extracted with EtOAc (3 × 75 mL). The combined organic layers were dried over Na_2_SO_4_, concentrated on a rotary evaporator, and purified by flash chromatography (SiO_2_, 20–40% EtOAc/Hexanes) to provide diester **23** as an amorphous white solid (141 mg, 85%): mp 47–51 °C; ^1^H NMR (CDCl_3_, 400 MHz) δ 3.67 (s, 3H), 3.67 (s, 3H), 3.61–3.57 (m, 1H), 2.31 (dt, *J* = 11.2, 7.5 Hz, 4H), 1.71–1.58 (m, 4H), 1.50–1.35 (m, 7H), 1.32–1.24 (m, 12H); ^13^C NMR (CDCl_3_, 100 MHz) δ 174.5, 174.3, 71.8, 51.6, 51.6, 37.7, 37.2, 34.2, 34.2, 29.8, 29.7, 29.5, 29.3, 29.3, 25.8, 25.3, 25.1, 25.0; IR (film) ν_max_ 3532, 2911, 2850, 1733, 1717, 1246, 1206, 1174 cm^−1^; HRMS (DART-TOF) *m/z* 331.2488 (C_18_H_34_O_5_ + H^+^ requires 331.2484).

### Dimethyl (*E*)-6-((3-(4-((*tert*-Butyldimethylsilyl)oxy)phenyl)acryloyl)oxy)hexadecaned-ioate (24)

A solution of diester **23** (600 mg, 1.82 mmol) in dry CH_2_Cl_2_ (18.0 mL) at 23 °C was treated sequentially with protected coumaric acid **S8**^50,51^ (811 mg, 2.91 mmol), DMAP (44.4 mg, 0.363 mmol), and DCC (563 mg, 2.73 mmol). The resulting solution was warmed to 40 °C and stirred at 40 °C for 20 h. After 20 h, the reaction mixture was diluted with Et_2_O (50 mL), filtered through Celite, concentrated on a rotary evaporator, and purified by flash chromatography (SiO_2_, 15–30% Et_2_O/Hexanes) to provide coumaric ester **24** as a clear, colorless oil (743 mg, 69%): ^1^H NMR (CDCl_3_, 500 MHz) δ 7.61 (d, *J* = 15.9 Hz, 1H), 7.42 (d, *J* = 8.6 Hz, 2H), 6.83 (d, *J* = 8.6 Hz, 2H), 6.29 (d, *J* = 16.0 Hz, 1H), 4.99 (qd, *J* = 7.2, 5.2 Hz, 1H), 3.65 (s, 3H), 3.65 (s, 3H), 2.29 (dt, *J* = 10.5, 7.6 Hz, 4H), 1.71–1.51 (m, 8H), 1.43–1.24 (m, 14H), 0.98 (s, 9H), 0.21 (s, 6H); ^13^C NMR (CDCl_3_, 125 MHz) δ 174.5, 174.2, 167.3, 157.9, 144.3, 129.8, 128.0, 120.6, 116.4, 74.0, 51.6, 51.6, 34.4, 34.2, 34.1, 29.6, 29.6, 29.5, 29.3, 29.3, 25.8, 25.8, 25.8, 25.5, 25.1, 25.0, 18.4, –4.3; IR (film) ν_max_ 2929, 2856, 1737, 1706, 1634, 1509, 1254, 1164, 908, 836, 781 cm^−1^; HRMS (DART-TOF) *m/z* 591.3795 (C_33_H_54_O_7_Si + H^+^ requires 591.3717).

### 1,16-Dihydroxyhexadecan-6-yl (*E*)-3-(4-((*tert*-Butyldimethylsilyl)oxy)phenyl)acrylate (S9)

A solution of coumaric ester **24** (742 mg, 1.26 mmol) in dry Et_2_O (15.0 mL) at 23 °C was treated with solid LiBH_4_ (222 mg, 10.0 mmol). The resulting solution was stirred at 23 °C for 3 h. After 3 h, the reaction mixture was treated dropwise with sat. NaHCO_3_(aq) (15 mL). Stirring was continued until off-gassing ceased. The biphasic mixture, thus obtained, was extracted with Et_2_O (3 × 10 mL) and the combined organic layers were dried over Na_2_SO_4_, concentrated on a rotary evaporator, and purified by flash chromatography (SiO_2_, 20–60% EtOAc/Hexanes) to provide a clear, colorless oil containing the product (**S9**, 432 mg, 64%) and over-reduced byproduct (**S10**, 109 mg) as an inseparable mixture. For **S9**: ^1^H NMR (CDCl_3_, 500 MHz) δ 7.61 (d, *J* = 15.9 Hz, 1H), 7.42 (d, *J* = 8.6 Hz, 2H), 6.83 (d, *J* = 8.6 Hz, 2H), 6.30 (d, *J* = 15.9 Hz, 1H), 5.01 (qd, *J* = 7.4, 5.1 Hz, 1H), 3.63 (t, *J* = 6.6 Hz, 4H), 1.66–1.45 (m, 8H), 1.44–1.18 (m, 18H), 0.98 (s, 9H), 0.21 (s, 6H); ^13^C NMR (CDCl_3_, 125 MHz) δ 173.0, 167.4, 157.9, 144.3, 129.8, 120.6, 116.4, 74.2, 63.2, 62.9, 34.5, 34.4, 32.9, 32.8, 29.7, 29.6, 29.6, 29.6, 29.5, 25.9, 25.8, 25.8, 25.7, 25.5, 25.2, –4.2; HRMS (DART-TOF) *m/z* 535.3895 (C_31_H_54_O_5_Si + H^+^ requires 535.3819).

### 1,16-Dihydroxyhexadecan-6-yl (*E*)-3-(4-Hydroxyphenyl)acrylate (S11)

A solution of diol **S9** (307 mg, 0.574 mmol) and over-reduced byproduct **S10** (78 mg) in THF (13.3 mL) at 23 °C was treated with a 1 M solution of TBAF in THF (1.08 mL, 1.08 mmol). The resulting solution was stirred for 5 min. After 5 min, the reaction mixture was treated with 1 M HCl(aq) (10 mL) and extracted with EtOAc (3 × 10 mL). The combined organic layers were dried over Na_2_SO_4_, concentrated on a rotary evaporator, and purified by flash chromatography (SiO_2_, 80% EtOAc/Hexanes) to provide a clear, colorless oil containing the triol product (**S11**, 217 mg, 90%) and over-reduced byproduct (**S12**, 55 mg) as an inseparable mixture. For **S11**: ^1^H NMR (CDCl_3_, 500 MHz) δ 7.61 (d, *J* = 15.9 Hz, 1H), 7.41 (d, *J* = 8.7 Hz, 2H), 6.84 (d, *J* = 8.6, 2H), 6.47 (bs, 1H), 6.28 (d, *J* = 15.9 Hz, 1H), 5.01 (tt, *J* = 7.5, 5.0 Hz, 1H), 3.67–3.60 (m, 4H), 1.71–1.48 (m, 8H), 1.48–1.15 (m, 18H); ^13^C NMR (CDCl_3_, 125 MHz) δ 167.7, 158.3, 144.6, 130.1, 127.1, 116.1, 115.9, 74.3, 63.3, 62.9, 34.5, 34.4, 32.9, 32.6, 29.6, 29.6, 29.6, 29.5, 29.5, 25.8, 25.6, 25.4, 25.2; HRMS (DART-TOF) *m/z* 419.2881 (C_25_H_40_O_5_ – H^+^ requires 419.2798).

### 1,16-Dioxohexadecan-6-yl (*E*)-3-(4-Hydroxyphenyl)acrylate (25)

A solution of triol **S11** (94.2 mg, 0.224 mmol) and over-reduced byproduct **S12** (23.8 mg) in CH_2_Cl_2_ (2.56 mL) at 0 °C was treated sequentially with Et_3_N (1.30 mL) and a solution of SO_3_ ∙ py (267 mg, 1.68 mmol) in DMSO (1.77 mL). The resulting solution was stirred at 0 °C for 2.5 h. After 2.5 h, the reaction mixture was treated with 1 M HCl(aq) (5 mL) and extracted with EtOAc (3 × 5 mL). The combined organic layers were dried over Na_2_SO_4_, concentrated on a rotary evaporator, and purified by PTLC (SiO_2_, 50% EtOAc/Hexanes) to provide dialdehyde **25** as a clear, colorless oil (54.2 mg, 58%): ^1^H NMR (DMSO-*d*_6_, 500 MHz) δ 10.00 (s, 1H), 9.65 (dt, *J* = 3.2, 1.6 Hz, 2H), 7.57–7.52 (m, 3H), 6.79 (d, *J* = 8.7 Hz, 2H), 6.38 (d, *J* = 16.0 Hz, 1H), 4.93–4.88 (m, 1H), 2.43 (td, *J* = 7.2, 1.6 Hz, 2H), 2.39 (td, *J* = 7.3, 1.7 Hz, 2H), 1.58–1.47 (m, 8H), 1.33–1.24 (m, 14H); ^13^C NMR (CDCl_3_, 125 MHz) δ 203.4, 202.9, 167.5, 158.0, 144.5, 130.1, 127.3, 116.0, 116.0, 74.1, 44.0, 43.9, 34.4, 34.2, 29.6, 29.6, 29.4, 29.4, 29.3, 25.5, 25.1, 22.2, 22.1; IR (film) ν_max_ 3347, 2926, 2854, 1704, 1603, 1585, 1514, 1261, 1164, 983, 832 cm^−1^; HRMS (DART-TOF) *m/z* 417.2740 (C_25_H_36_O_5_ + H^+^ requires 417.2641).

### Sporopollenin-inspired polymer synthesis via crosslinking

To a solution of 5.00 wt% PVA in DMSO (1.00 mL, 1.14 mmol) was added an appropriate amount of dialdehyde (*vide supra*) to achieve the desired DOC at 23 °C. The suspension was heated to 60 °C in a sealed vial until complete dissolution of the dialdehyde was achieved (approx. 5 min). To this warm solution was added 0.10 mL of acid catalyst solution containing 7.50 v/v% AcOH and 2.50 v/v% H_2_SO_4_ in DMSO. The resulting solution was vigorously homogenized and poured into silicone molds. Curing of this solution at 55–60 °C at 760 torr for 90–120 min under an atmosphere of air, then at 40 °C at 250 torr for 72 h under an atmosphere of air provided crosslinked polymers which were subsequently washed by submerging sequentially in distilled H_2_O (10 min), sat. NaHCO_3_(aq) (10 min), and distilled H_2_O again (2 h). Drying of the resultant materials under ambient conditions for 24 h provided sporopollenin-inspired polymers which were utilized, without further treatment, for chemical, physical, and thermal analyses.

### Solvolysis studies

One disk of sporopollenin-inspired polymer (*vide supra*) of known mass was placed in 10 mL of solvent and allowed to shake (150 rpm) at 23 °C for 24 h. The polymer disk was then dried at 40 °C and 250 torr for 24 h unless otherwise noted, allowed to equilibrate under ambient conditions for 24 h, then weighed to determine total mass loss during solvolysis. All observed mass losses, with the exception of the conc. H_2_SO_4_ sample, are below the mass fraction attributed to volatile low molecular weight compounds (i.e., residual solvent) in the starting samples as observed by TGA (*vide infra*).

### Degradative thioacidolysis/liquid chromatography-high resolution mass spectrometry (DT/LC-HRMS)

A suspension of authentic sporopollenin or synthetic sporopollenin analogue (4.0 mg) in dry 1,4-dioxane (9.0 mL) was treated sequentially with ethane thiol (1.0 mL, 14 mmol) and >46.5% boron trifluoride diethyl etherate (0.25 mL, 0.05 mmol) at 23 °C. The resulting suspension was sealed and heated to 100 °C for 4 h. After 4 h, the reaction mixture was cooled to 23 °C, treated dropwise with sat. NaHCO_3_(aq) to achieve a pH of approximately 4, and extracted with CHCl_3_ (3 × 5 mL). The combined organic layers were dried over Na_2_SO_4_ and concentrated under a stream of nitrogen. The crude product was dissolved in CHCl_3_, filtered, and submitted for LC-HRMS analysis^35^.

### Durometer hardness

Durometer hardness measurements were conducted by Element Materials Technology in accordance with ASTM D2240 (excepting sample geometry) with conditioning at laboratory conditions of 23 ± 2 °C and 50 ± 10% relative humidity. Samples were disks approximately 1.4 mm in thickness and 3.5 mm in diameter. Due to the small sample size, one reading was taken from each individual sample. Hardness reported on the Shore D scale.

### Compression testing

Compression testing was conducted in triplicate according to the following procedure: We fabricated the testing samples in a disk shape with a diameter *D* of around 3.7 mm and a thickness *H* of around 1.6 mm. The disk-shaped sample was compressed using a mechanical tester from Zwick/Roell company up to its nominal strain of 80% and subsequently unloaded to its original state. The loading speed was set as 1 mm/min. The measured nominal stress *s* versus nominal strain ε of the sample can be calculated via *s* = *F*/*A* and *ε* = ∆/*H*, where *F* is the measured force, *A* = *πD*^2^/4 is the cross-sectional area of the sample with *D* as the diameter of the sample, ∆ is the loading displacement, and *H* is the thickness of the sample. The elastic modulus of the sample was calculated via *E* *=* *ds*/*dε*|_*ε*=0_). The hysteresis ratio was calculated by $h={\int }_{0}^{{\varepsilon }_{{\max }}}{sd}\varepsilon$, where *ε*_*max*_ is the maximum nominal strain during the cyclic compressive loading.

### Tensile testing

Tensile testing was conducted in triplicate according to the following procedure: We fabricated the testing samples in a strip shape with a width *W* of around 8.6 mm, a thickness *T* of around 1.6 mm, and a height *H* of around 10 mm. A monotonic tensile load was applied on the strip-shaped sample using a mechanical tester from Zwick/Roell company up to the rupture of the sample. The loading speed was set as 1 mm/min. The measured nominal stress *s* versus nominal strain ε of the sample can be calculated via *s* = *F*/*A* and *ε* = ∆/*H*, where *F* is the measured force, *A* = *WT* is the cross-sectional area of the sample with *W* as the width of the sample and *T* as the thickness of the sample, ∆ is the loading displacement, and *H* is the height of the sample. We also performed a cyclic tensile loading on the sample at the same loading speed of 1 mm/min, measuring its hysteresis at various applied strains under tensile loading.

### Swelling ratio

The measurement of swelling ratio was conducted in triplicate according to the following procedure: We fabricated the testing samples in a disc shape with a diameter *D* of around 3.7 mm and a thickness *H* of around 1.6 mm. Both the volume and weight of the sample were measured before and after immersing in a deionized water, measuring the volume swelling ratios and the weight swelling ratios, respectively.

### Thermogravimetric analysis (TGA)

Thermogravimetric analysis was conducted in triplicate by Element Materials Technology in accordance with Element New Berlin Procedure PA-04 with all polymer samples disclosed herein. Samples were heated from 20 °C to 650 °C at a rate of 20 °C/min under an atmosphere of nitrogen, cooled to 500 °C, exposed to air, then heated to 800 °C at a rate of 20 °C/min. Three mass loss events were observed. The first, observed at 184–236 °C in nitrogen, corresponds to evolution of loss of low molecular weight (i.e., solvents). The second, observed at 440–464 °C in nitrogen, corresponds to decomposition of the base polymer. The third, observed at 519–540 °C in air, corresponds to combustion which left behind a particulate carbonaceous residue.

### Differential scanning calorimetry (DSC)

Differential scanning calorimetry was conducted in triplicate by Element Materials Technology in accordance with Element New Berlin Procedure PA-06 with synthetic sporopollenin analogue **15**. A three-step methodology was employed in which samples were heated from –60 °C to 275 °C, control cooled to –60 °C, then heated to 300 °C under an atmosphere of nitrogen. No significant thermal features were observed below 110 °C. Above 110 °C, volatilization of residual low molecular weight compounds (i.e., solvents, observed via TGA) interfered with observations. Subsequent modulated DSC focused below 110 °C was performed by heating samples from –60 °C to 110 °C at an underlying heating rate of 3 °C/min which was modulated ±1 °C every 60 s.

## Supplementary information

Weng_PR File

Supplementary Material

## Data Availability

Additional experimental details including: supplementary methods, Supplementary Notes, Supplementary Tables S1–S9, and Supplementary Figs. S1–S62 are available in the Supplementary Information. The raw data that support the findings of this study are available from the corresponding author upon reasonable request. Correspondence and request for materials should be addressed to J.-K.W.
