# Supplementary material for: Sporopollenin-inspired design and synthesis of robust polymeric materials
Source: Commun Chem. 2022 Sep 12;5:110. doi: 10.1038/s42004-022-00729-w (PMC9814627; doi:10.1038/s42004-022-00729-w)
Supplement: Supplementary file 2 — Supplementary Material [file 42004_2022_729_MOESM2_ESM.pdf]

Supplementary Materials for

Sporopollenin-inspired design and synthesis of robust polymeric materials

Christopher M. Glinkerman, Shaoting Lin, Jiahua Ni, Fu-Shuang Li, Xuanhe Zhao, Jing-Ke Weng*

*Correspondence to: wengj@wi.mit.edu

This PDF file includes:

Supplementary Methods
Supplementary Note 1: Sample Degree of Crosslinking (DOC) Calculation
Supplementary Tables
Table S1. DOC results for 50 mg PVA crosslinked by dialdehyde **15**
Table S2. Synthetic Sporopollenin Analogue Numbering Key
Table S3. Chemical stability of synthetic sporopollenin polymer **15**
Table S4. Durometer hardness (Shore D) of synthetic sporopollenin analogues
Table S5. Moduli (MPa) of synthetic sporopollenin analogues
Table S6. Hysteresis ratio (dimensionless) of synthetic sporopollenin analogues
Table S7. Volume swelling ratios (dimensionless) of synthetic sporopollenin analogues
Table S8. Weight swelling ratios (dimensionless) of synthetic sporopollenin analogues
Table S9. Thermogravimetric analysis of synthetic sporopollenin analogues
Supplementary Figures
Figure S1. The ^{1}H NMR spectrum of compound **S6**
Figure S2. The ^{1}H NMR spectrum of compound **S7**
Figure S3. The ^{1}H NMR spectrum of compound **21**
Figure S4. The ^{1}H NMR spectrum of compound **22**
Figure S5. The ^{1}H NMR spectrum of compound **23**
Figure S6. The ^{1}H NMR spectrum of compound **24**
Figure S7. The ^{1}H NMR spectrum of compounds **S9** and **S10**
Figure S8. The ^{1}H NMR spectrum of compounds **S11** and **S12**
Figure S9. The ^{1}H NMR spectrum of compound **25**
Figure S10. The ^{13}C NMR spectrum of compound **25**
Figure S11. The ^{13}C MAS ssNMR spectrum of authentic *P. rigida* sporopollenin
Figure S12. The ^{13}C MAS ssNMR spectrum of simplified sporopollenin analogue **15**
Figure S13. The ^{13}C MAS ssNMR spectrum of synthetic linker sporopollenin analogue **26**
Figure S14. Overlaid ^{13}C MAS ssNMR spectra of authentic *P. rigida* sporopollenin (blue), simplified sporopollenin analogue **15** (green), and synthetic linker sporopollenin analogue **26** (red)
Figure S15. The IR spectrum of authentic *P. rigida* sporopollenin
Figure S16. The IR spectrum of simplified sporopollenin analogue **15**
Figure S17. The IR spectrum of synthetic linker sporopollenin analogue **26**
Figure S18. Overlaid IR spectra of authentic *P. rigida* sporopollenin (blue), simplified sporopollenin analogue **15** (green), and synthetic linker sporopollenin analogue **26** (red)

Figure S19. The base peak chromatograms of thioacidolyzed authentic *P. rigida* sporopollenin (red), simplified sporopollenin analogue **15** (blue), and synthetic linker sporopollenin analogue **26** (black)
Figure S20. The extracted-ion chromatograms of thioacidolyzed authentic *P. rigida* sporopollenin (red), simplified sporopollenin analogue **15** (blue), and synthetic linker sporopollenin analogue **26** (black)
Figure S21. Compressive stress-strain curve for synthetic sporopollenin analogue **6**
Figure S22. Compressive stress-strain curve for synthetic sporopollenin analogue **7**
Figure S23. Compressive stress-strain curve for synthetic sporopollenin analogue **8**
Figure S24. Compressive stress-strain curve for synthetic sporopollenin analogue **9**
Figure S25. Compressive stress-strain curve for synthetic sporopollenin analogue **10**
Figure S26. Compressive stress-strain curve for synthetic sporopollenin analogue **11**
Figure S27. Compressive stress-strain curve for synthetic sporopollenin analogue **12**
Figure S28. Compressive stress-strain curve for synthetic sporopollenin analogue **13**
Figure S29. Compressive stress-strain curve for synthetic sporopollenin analogue **14**
Figure S30. Compressive stress-strain curve for synthetic sporopollenin analogue **15**
Figure S31. Compressive stress-strain curve for synthetic sporopollenin analogue **16**
Figure S32. Compressive stress-strain curve for synthetic sporopollenin analogue **17**
Figure S33. Compressive stress-strain curve for synthetic sporopollenin analogue **18**
Figure S34. Compressive stress-strain curve for synthetic sporopollenin analogue **26**
Figure S35. Relationship between compressive modulus and linker carbon count in simplified (black) and substituted (red) synthetic linker sporopollenin analogues
Figure S36. Relationship between compressive modulus and linker density in simplified (black) and substituted (red) synthetic linker sporopollenin analogues
Figure S37. Relationship between compressive modulus and PVA MW_{avg} in simplified (black) and substituted (red) synthetic linker sporopollenin analogues
Figure S38. Relationship between compressive hysteresis ratio and linker carbon count in simplified (black) and substituted (red) synthetic linker sporopollenin analogues
Figure S39. Relationship between compressive hysteresis ratio and linker density in simplified (black) and substituted (red) synthetic linker sporopollenin analogues
Figure S40. Relationship between compressive hysteresis ratio and PVA MW_{avg} in simplified (black) and substituted (red) synthetic linker sporopollenin analogues
Figure S41. Tensile stress-strain curve for synthetic sporopollenin analogue **12** at 1.0 (red) and 0.1 min^{-1} (black) loading rates
Figure S42. Tensile stress-strain curve for synthetic sporopollenin analogue **12** with cyclic (red) and monotonic (black) loading
Figure S43. Tensile stress-time relaxation curve for synthetic sporopollenin analogue **12**
Figure S44. The TGA thermogram for synthetic sporopollenin analogue **6**
Figure S45. The TGA thermogram for synthetic sporopollenin analogue **7**
Figure S46. The TGA thermogram for synthetic sporopollenin analogue **8**
Figure S47. The TGA thermogram for synthetic sporopollenin analogue **9**
Figure S48. The TGA thermogram for synthetic sporopollenin analogue **10**
Figure S49. The TGA thermogram for synthetic sporopollenin analogue **11**
Figure S50. The TGA thermogram for synthetic sporopollenin analogue **12**
Figure S51. The TGA thermogram for synthetic sporopollenin analogue **13**

Figure S52. The TGA thermogram for synthetic sporopollenin analogue **14**
Figure S53. The TGA thermogram for synthetic sporopollenin analogue **15**
Figure S54. The TGA thermogram for synthetic sporopollenin analogue **16**
Figure S55. The TGA thermogram for synthetic sporopollenin analogue **17**
Figure S56. The TGA thermogram for synthetic sporopollenin analogue **18**
Figure S57. The TGA thermogram for synthetic sporopollenin analogue **26**
Figure S58. The DSC thermogram for synthetic sporopollenin analogue **15**, first replicate
Figure S59. The DSC thermogram for synthetic sporopollenin analogue **15**, second replicate
Figure S60. The DSC thermogram for synthetic sporopollenin analogue **15**, third replicate
Figure S61. The DSC thermogram for synthetic sporopollenin analogue **15**, all replicates
Figure S62. The modulated DSC thermogram for synthetic sporopollenin analogue **15**

Supplementary Methods

All reactions were performed under nitrogen unless otherwise noted. 1,8-octanediol (TCI, >99%), 1,10-decanediol (TCI, >95%), 1,12-dodecanediol (Acrōs, 99%), 1,14-tetradecanediol (Combi-Blocks, 97%), 1,16-hexadecanediol (TCI, >95%), Dess-Martin periodinane (Oakwood, 95%), polyvinyl alcohol ($M_{w,avg}$ = 31-50k, Sigma Aldrich, 98–99% hydrolyzed), polyvinyl alcohol ($M_{w,avg}$ = 85-124k, Sigma Aldrich, >99% hydrolyzed), polyvinyl alcohol ($M_{w,avg}$ = 89-98k, Sigma Aldrich, >99% hydrolyzed), polyvinyl alcohol ($M_{w,avg}$ = 146-186k, Sigma Aldrich >99% hydrolyzed), cyclohexanone (Acrōs, >99%), cyclohexylamine (TCI, >99%), *n*-butyllithium (Acrōs, 2.45 M), diisopropylamine (Spectrum, >99%), 11-bromo-1-undecene (Oakwood, 95%), sodium (meta)periodate (BTC, 98%), potassium permanganate (J.T. Baker, 99.4%), methyl iodide (Alfa Aesar, >98%), potassium carbonate (Sigma Aldrich, 99%), *meta*-chloroperbenzoic acid (Sigma Aldrich, <77%), sodium methoxide (Acrōs, >99%), 4-dimethylaminopyridine (Acrōs, 99%), *N,N'*-dicyclohexylcarbodiimide (TCI, >98%), lithium borohydride (Strem, 95%), tetrabutylammonium fluoride (Oakwood, 1 M in THF), triethylamine (Acrōs, 99%), sulfur trioxide pyridine complex (TCI, >95%), coumaric acid (TCI, >98%), *tert*-butyldimethylsilyl chloride (Chem-Impex, 99%), imidazole (Sigma Aldrich, 99%), ethane thiol (Sigma Aldrich, 97%), boron trifluoride diethyl etherate (Sigma Aldrich, >46.5% BF_3 basis), acetic acid (Spectrum, >99.7%), concentrated sulfuric acid (EMD Millipore, 95%), 4 Å molecular sieves (Sigma Aldrich), ammonium chloride (Mallinckrodt, 99.5%), sodium sulfate (Sigma Aldrich, >99%), Celite (Fisher), sodium bicarbonate (Mallinckrodt, >99.7%), conc. hydrochloric acid (Macron, 36.5–38.0%), sodium hydroxide (AmericanBio, 97%), sodium chloride (EMD Millipore, 99.5%), dicholoromethane (Fisher, >99.5%), dimethylsulfoxide (Fisher, >99.9%), diethyl ether (Fisher, >99.9%), tetrahydrofuran (Acrōs, >99.5%, extra dry, stabilized with BHT), acetone (Fisher, >99.5%), water (distilled), petroleum ether (Fisher, 36–60 °C Fraction), hexanes (Fisher, 98.5%), ethyl acetate (Fisher, 99.5%), dimethylformamide (Acrōs, 99.8%), chloroform (EMD Millipore, >99.8%), methanol (EMD Millipore, 99.9%), and dioxane (Acrōs, 99.8%) were used as received without further purification unless otherwise noted. Column chromatography was conducted using Silicycle SiliaFlash P60 SiO_2 (40–63 µm). Preparative TLC was conducted using Millipore SiO_2 60 F_{254} PTLC (0.5 mm). Analytical TLC was conducting using Millipore SiO_2 60 F_{254} TLC (0.250 mm) plates. Silicone molds for polymer curing were obtained from MiniatureSweet ("Small Geometry Silicone Mold").

Melting points were obtained using a Mel-Temp II apparatus in open capillaries and are uncorrected. Solution state ^1H and ^{13}C NMR spectra were obtained using either a Bruker Avance Neo 400 MHz spectrometer equipped with a 5 mm BBFO SmartProbe or a Bruker Avance Neo 500 MHz spectrometer equipped with a 5 mm liquid nitrogen cooled Prodigy BBO cryoprobe using either $CDCl_3$ (Cambridge Isotope Laboratories, 99.8%D) or DMSO-d_6 (Cambridge Isotope Laboratories, 99.9%D) as solvents. ^{13}C MAS ssNMR spectra were obtained on a Bruker Avance Neo 500 MHz spectrometer equipped with a 3.2 mm HX solids probe set to a MAS speed of 20 kHz. IR spectra were obtained using a Bruker Alpha 2 with a Platinum ATR accessory. High resolution mass spectrometric analysis was performed on either a JEOL AccuTOF-DART or a Thermo Fisher Scientific Q-Exactive benchtop Orbitrap. Durometer hardness testing was performed by Element Materials Technology with a Durometer Type D from Pacific Transducer Corporation. Thermogravimetric analysis was performed by Element Materials Technology with a TGA Q500 from TA Instruments. Differential scanning calorimetry was performed by Element Materials Technology with a DSC Q2000 from TA Instruments. Mechanical characterization was performed with a mechanical testing apparatus from Zwick/Roell company.

Supplementary Note 1: Sample Degree of Crosslinking (DOC) Calculation

Formula:

$$\frac{mass\ PVA}{1} \times \frac{mol\ PVA}{44\ g\ PVA} \times \frac{mol - OH}{mol\ PVA} \times \frac{mol\ crosslinker}{4\ mol - OH} \times \frac{g}{mo} \quad \begin{matrix} g\ crosslinker\ to\ achieve \\ 100\%\ theoretical\ DOC \end{matrix}$$

Sample Calculation:

If 1 mL of 5 wt% PVA in DMSO is to be 20% crosslinked by dialdehyde **15:**

$$0.05\ g\ PVA \times \frac{mol\ PVA}{44\ g} \times \frac{mol\ crosslinker}{4\ mol - OH} \times \frac{254.41\ g\ crosslinker}{mol\ crosslinker} \times 0.2 = 14.5\ mg\ crosslinker$$

Supplementary Tables

Table S1. DOC results for 50 mg PVA crosslinked by dialdehyde **15**

Theoretical DOC (%)	Mass of Linker (mg)
0	0.000
1	0.723
2	1.45
5	3.63
10	7.25
20	14.5
30	21.7
40	28.9
50	36.1

Table S2. Synthetic Sporopollenin Analogue Numbering Key

**Sporopollenin Analogue #**	**6**	**7**	**8**	**9**	**10**	**11**	**12**
Linker Carbon Count (#C)	16	16	16	8	10	12	14
Linker Density (%)	20	20	20	20	20	20	20
PVA MW_{avg} (g/mol)	166000	104500	40500	93500	93500	93500	93500
**Sporopollenin Analogue #**	**13**	**14**	**15**	**16**	**17**	**18**	**26**
Linker Carbon Count (#C)	16	16	16	16	16	16	16*
Linker Density (%)	5	10	20	30	40	50	20
PVA MW_{avg} (g/mol)	93500	93500	93500	93500	93500	93500	93500

* Indicates synthetic substituted crosslinker

Table S3. Chemical stability of synthetic sporopollenin polymer **15**

Solvent	Initial Mass (mg)	Final Mass (mg)	Mass Loss (%)	Notes:
CH_2Cl_2	29.28	26.84	8.334	N/A
Hexanes	28.75	26.86	6.574	N/A
Ethyl Acetate	27.34	25.63	6.255	N/A
Acetone	27.51	26.08	5.198	N/A
DMF	27.61	30.00	–8.656	48 h dry time
DMSO	27.10	32.02	–18.155	48 h dry time
H_2O	24.81	22.57	9.029	N/A
THF	26.46	25.13	5.026	N/A
$CHCl_3$	27.27	25.84	5.140	N/A
conc. H_2SO_4	27.11	N/A	N/A	Blackened and crumbling
5 N NaOH	27.39	29.03	–5.988	48 h dry time, add'l mass likely due to retained NaOH in sample

Table S4. Durometer hardness (Shore D) of synthetic sporopollenin analogues

**Sporopollenin Analogue #**	**6**	**7**	**8**	**9**	**10**	**11**	**12**
Sample 1 (Shore D)	67	76	67	70	69	85	77
Sample 2 (Shore D)	69	77	69	68	69	84	75
Sample 3 (Shore D)	69	78	68	66	69	83	75
Sample 4 (Shore D)	70	77	---	62	71	84	---
**Average (Shore D)**	**69**	**77**	**68**	**67**	**70**	**84**	**76**
**SD (Shore D)**	**1**	**1**	**1**	**3**	**1**	**1**	**1**
**Sporopollenin Analogue #**	**13**	**14**	**15**	**16**	**17**	**18**	**26**
Sample 1 (Shore D)	77	68	67	71	65	66	70
Sample 2 (Shore D)	77	68	69	73	70	66	69
Sample 3 (Shore D)	73	67	70	72	65	65	75
Sample 4 (Shore D)	---	---	68	73	70	63	70
**Average (Shore D)**	**76**	**68**	**69**	**72**	**68**	**65**	**71**
**SD (Shore D)**	**2**	**1**	**1**	**1**	**3**	**1**	**3**

Overall:

Average (Shore D): 71
SD (Shore D): 5

Table S5. Moduli (MPa) of synthetic sporopollenin analogues

**Sporopollenin Analogue #**	**6**	**7**	**8**	**9**	**10**	**11**	**12**
Sample 1 (MPa)	120	143	112	95	115	200	210
Sample 2 (MPa)	100	184	103	90	136	270	188
Sample 3 (MPa)	134	170	92	105	148	220	192
**Average**	**118**	**166**	**102**	**97**	**133**	**230**	**197**
**SD**	**17**	**21**	**10**	**8**	**17**	**36**	**12**
**Sporopollenin Analogue #**	**13**	**14**	**15**	**16**	**17**	**18**	**26**
Sample 1 (MPa)	158	105	156	157	120	86	90
Sample 2 (MPa)	111	107	160	135	106	100	86
Sample 3 (MPa)	131	87	175	116	118	95	116
**Average**	**133**	**100**	**164**	**136**	**115**	**94**	**97**
**SD**	**24**	**11**	**10**	**21**	**8**	**7**	**16**

Overall:

Average (MPa): 134

SD (MPa): 41

Table S6. Hysteresis ratio (dimensionless) of synthetic sporopollenin analogues

**Sporopollenin Analogue #**	**6**	**7**	**8**	**9**	**10**	**11**	**12**
Sample 1	0.54	0.62	0.53	0.48	0.53	0.62	0.58
Sample 2	0.54	0.62	0.55	0.49	0.53	0.60	0.60
Sample 3	0.59	0.63	0.54	0.47	0.52	0.59	0.59
**Average**	**0.56**	**0.63**	**0.54**	**0.48**	**0.52**	**0.60**	**0.59**
**SD**	**0.03**	**0.01**	**0.01**	**0.01**	**0.01**	**0.01**	**0.01**
**Sporopollenin Analogue #**	**13**	**14**	**15**	**16**	**17**	**18**	**26**
Sample 1	0.58	0.56	0.63	0.55	0.55	0.55	0.57
Sample 2	0.58	0.57	0.63	0.55	0.54	0.56	0.56
Sample 3	0.60	0.55	0.64	0.57	0.55	0.56	0.58
**Average**	**0.59**	**0.56**	**0.63**	**0.56**	**0.55**	**0.56**	**0.57**
**SD**	**0.01**	**0.01**	**0.01**	**0.01**	**0.01**	**0.01**	**0.01**

Overall:

Average (dimensionless): 0.53
SD (dimensionless): 0.15

Table S7. Volume swelling ratios (dimensionless) of synthetic sporopollenin analogues

**Sporopollenin Analogue #**	**6**	**7**	**8**	**9**	**10**	**11**	**12**
Sample 1	1.24	1.47	1.38	1.51	1.44	1.62	1.53
Sample 2	1.28	1.41	1.35	1.56	1.40	1.57	1.50
Sample 3	1.27	1.39	1.39	1.54	1.41	1.70	1.51
**Average**	**1.26**	**1.43**	**1.37**	**1.54**	**1.42**	**1.63**	**1.51**
**SD**	**0.02**	**0.04**	**0.02**	**0.02**	**0.02**	**0.07**	**0.02**
**Sporopollenin Analogue #**	**13**	**14**	**15**	**16**	**17**	**18**	**26**
Sample 1	2.11	1.78	1.69	1.18	1.15	1.21	1.42
Sample 2	1.97	1.66	1.73	1.17	1.10	1.16	1.28
Sample 3	1.93	1.76	1.66	1.12	1.28	1.18	1.48
**Average**	**2.00**	**1.74**	**1.69**	**1.16**	**1.18**	**1.19**	**1.39**
**SD**	**0.10**	**0.06**	**0.03**	**0.03**	**0.09**	**0.03**	**0.10**

Table S8. Weight swelling ratios (dimensionless) of synthetic sporopollenin analogues

**Sporopollenin Analogue #**	**6**	**7**	**8**	**9**	**10**	**11**	**12**
Sample 1	1.24	1.43	1.24	1.61	1.33	1.55	1.41
Sample 2	1.21	1.34	1.27	1.43	1.30	1.49	1.33
Sample 3	1.26	1.32	1.25	1.47	1.26	1.56	1.35
**Average**	**1.23**	**1.37**	**1.25**	**1.50**	**1.30**	**1.54**	**1.37**
**SD**	**0.03**	**0.06**	**0.02**	**0.09**	**0.03**	**0.04**	**0.04**

**Sporopollenin Analogue #**	**13**	**14**	**15**	**16**	**17**	**18**	**26**
Sample 1	1.74	1.57	1.59	1.09	1.06	1.09	1.34
Sample 2	1.76	1.57	1.58	1.11	0.95	1.08	1.26
Sample 3	1.65	1.57	1.53	1.09	1.04	1.13	1.28
**Average**	**1.71**	**1.57**	**1.57**	**1.10**	**1.02**	**1.10**	**1.30**
**SD**	**0.06**	**0.00**	**0.03**	**0.01**	**0.06**	**0.02**	**0.04**

Table S9. Thermogravimetric analysis of synthetic sporopollenin analogues

**Sporopollenin Analogue #**	**6**	**7**	**8**	**9**	**10**	**11**	**12**
Loss 1 (%,°C)	33.4, 208	33.3, 217	34.4, 210	38.1, 211	37.2, 215	35.0, 218	34.9, 226
Loss 2 (%,°C)	55.6, 454	55.7, 455	53.8, 455	49.3, 446	50.1, 451	50.8, 452	51.8, 451
Loss 3 (%,°C)	9.7, 540	9.6, 535	10.4, 542	11.0, 532	11.1, 530	11.4, 534	10.8, 519
Residue (%)	1.3	1.5	1.3	1.6	1.6	2.7	2.5

**Sporopollenin Analogue #**	**13**	**14**	**15**	**16**	**17**	**18**	**26**
Loss 1 (%,°C)	37.9, 205	37.0, 210	35.6, 203	32.5, 220	30.6, 226	31.6, 218	32.3, 189
Loss 2 (%,°C)	50.1, 446	50.6, 450	53.2, 444	56.8, 457	60.8, 464	61.7, 463	56.5, 450
Loss 3 (%,°C)	10.6, 535	10.7, 535	9.0, 522	9.6, 540	7.9, 544	6.0, 548	9.8, 530
Residue (%)	1.4	1.6	2.2	1.1	0.7	0.7	1.3

Overall:

Average Loss 1 (%, °C): 34.6, 213
SD (%, °C): 2.4, 9.8

Average Loss 2 (%, °C): 54.1, 453
SD (%, °C): 4.0, 5.9

Average Loss 3 (%, °C): 9.8, 535
SD (%, °C): 1.4, 8.0

Average Residue (%): 1.5
SD (%): 0.6

Supplementary Figures

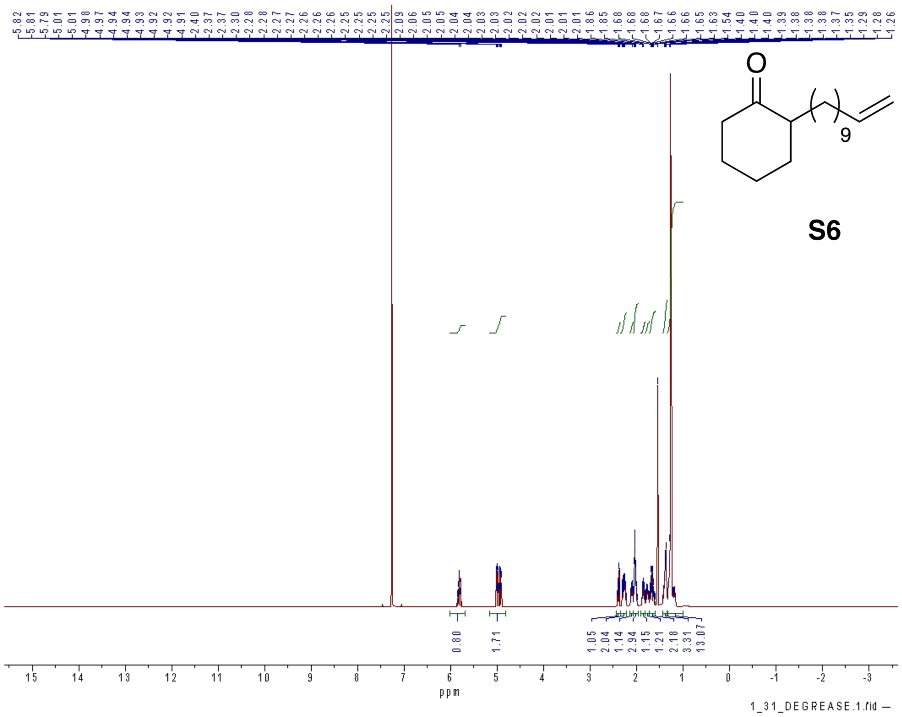

Figure S1. The ^{1}H NMR spectrum of compound **S6**

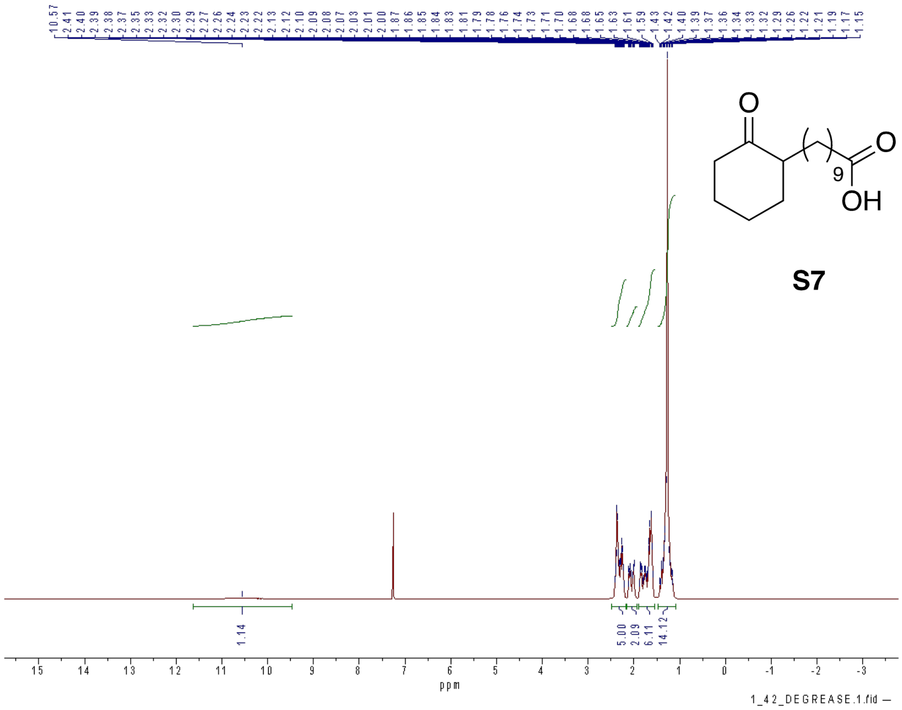

Figure S2. The ^{1}H NMR spectrum of compound **S7**

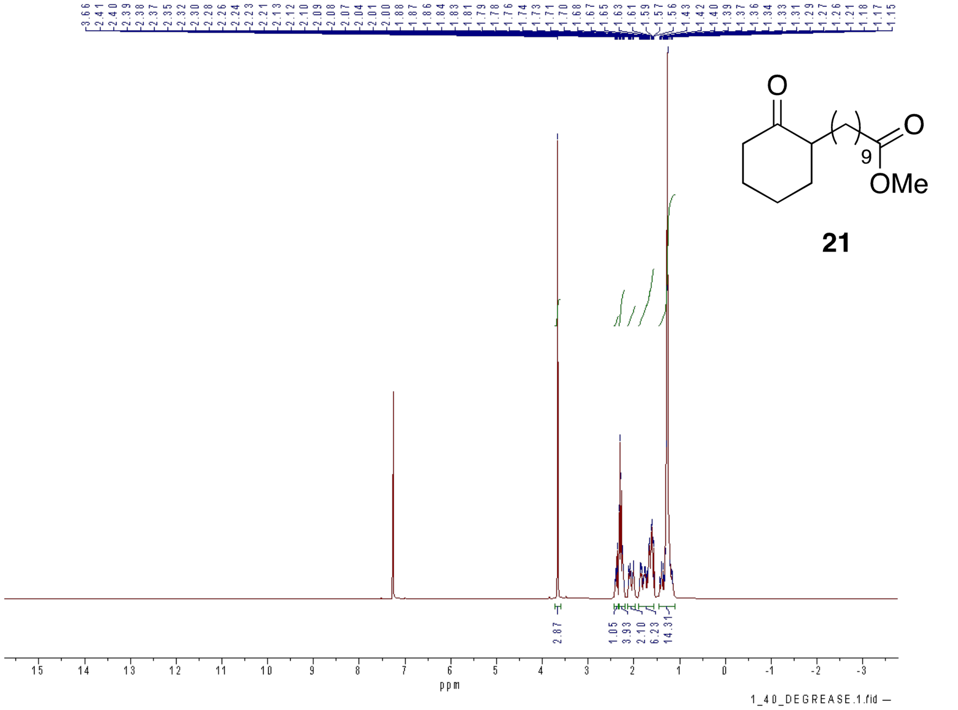

Figure S3. The ^{1}H NMR spectrum of compound **21**

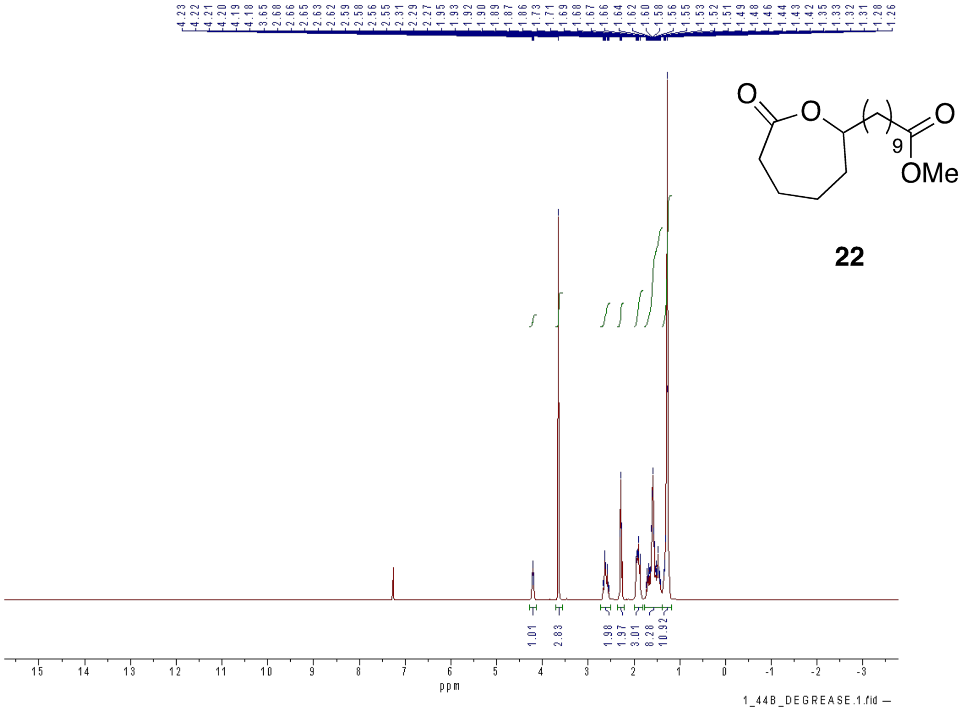

Figure S4. The ^{1}H NMR spectrum of compound **22**

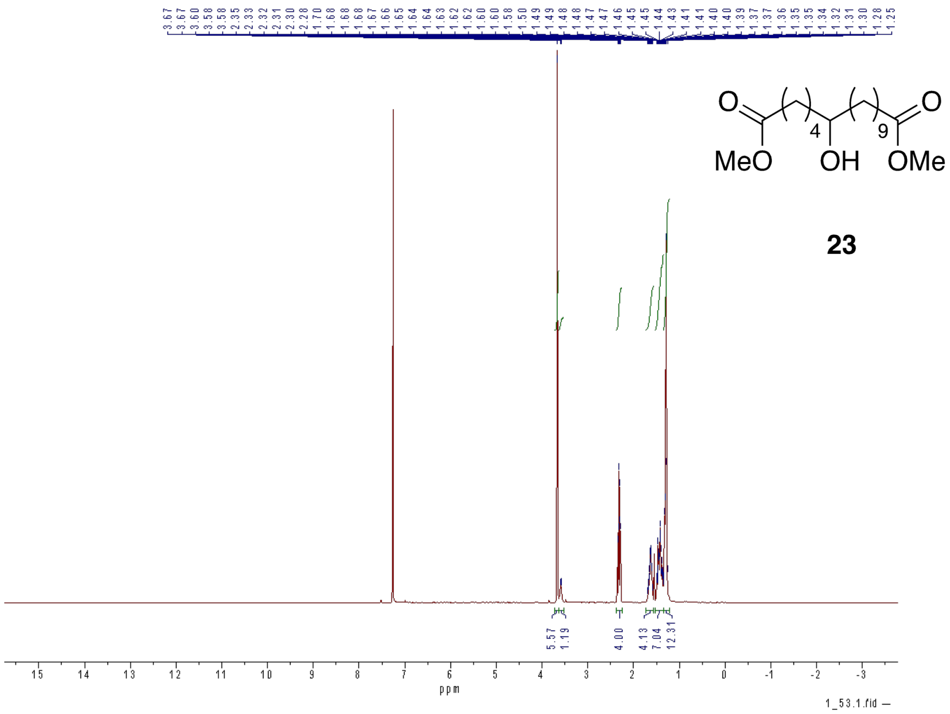

Figure S5. The ^{1}H NMR spectrum of compound **23**

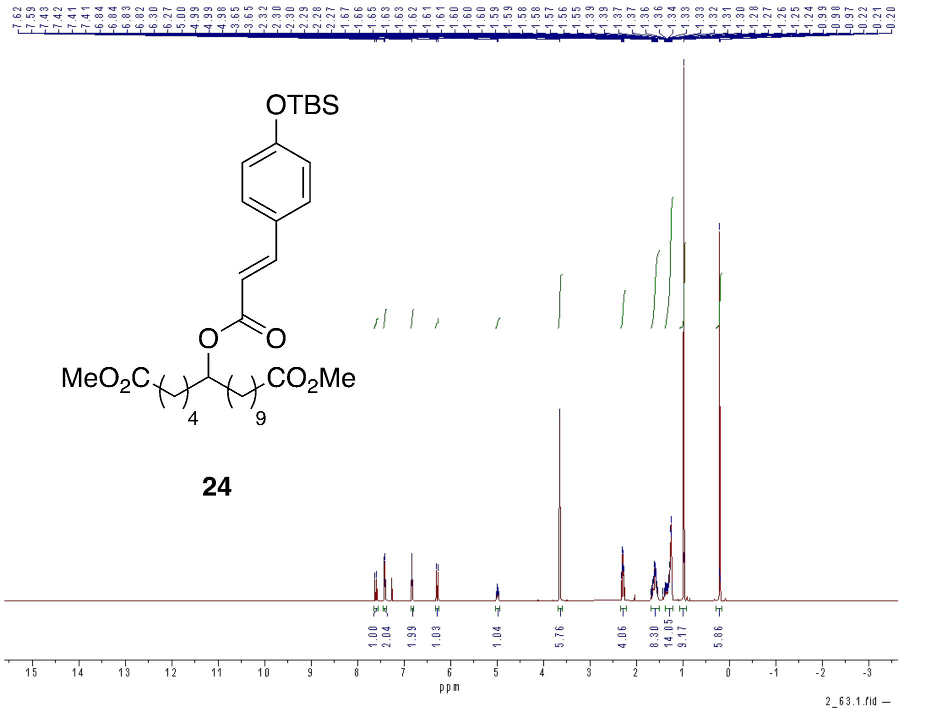

Figure S6. The ^{1}H NMR spectrum of compound **24**

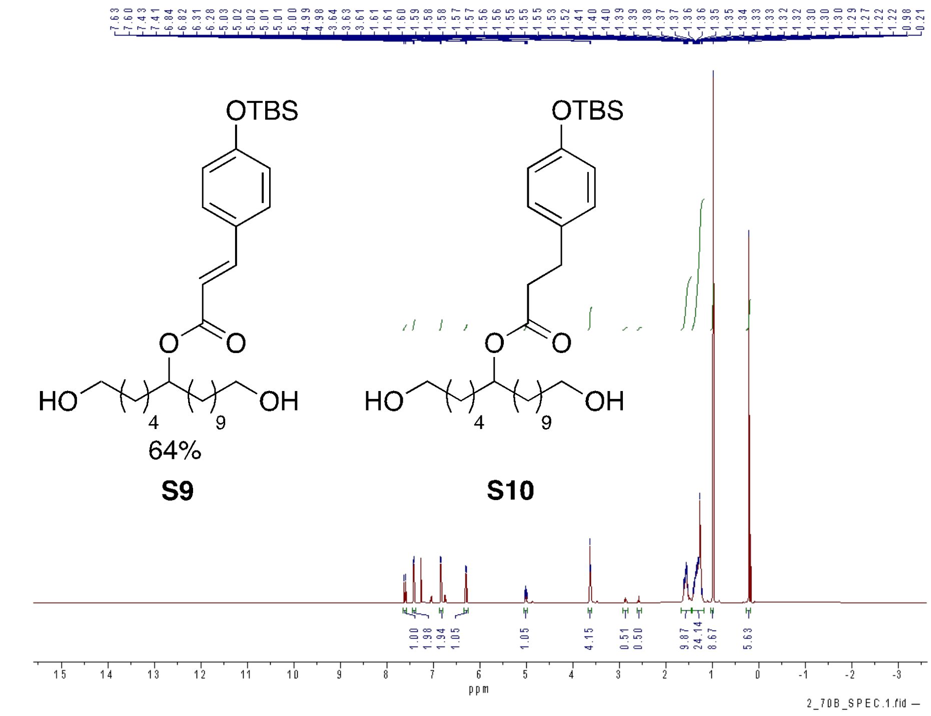

Figure S7. The ^{1}H NMR spectrum of compounds **S9** and **S10**

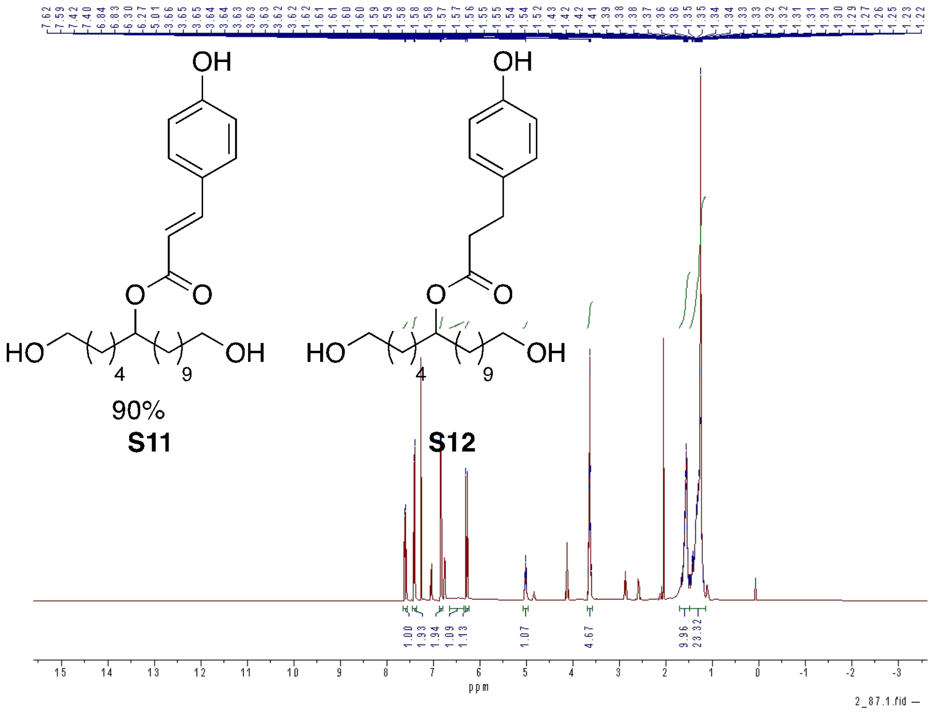

Figure S8. The ^{1}H NMR spectrum of compounds **S11** and **S12**

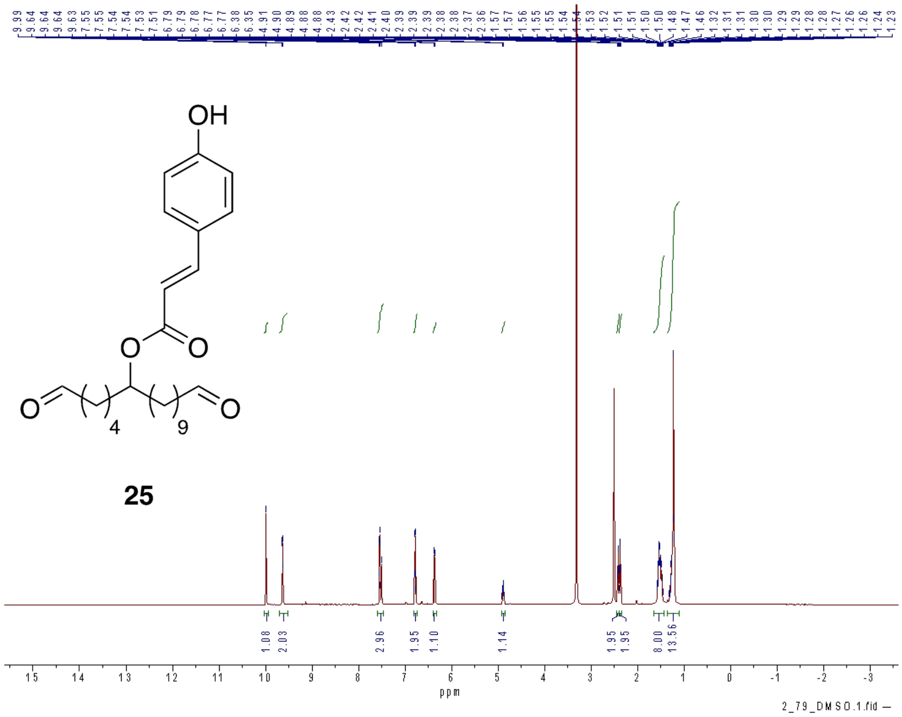

Figure S9. The ^{1}H NMR spectrum of compound **25**

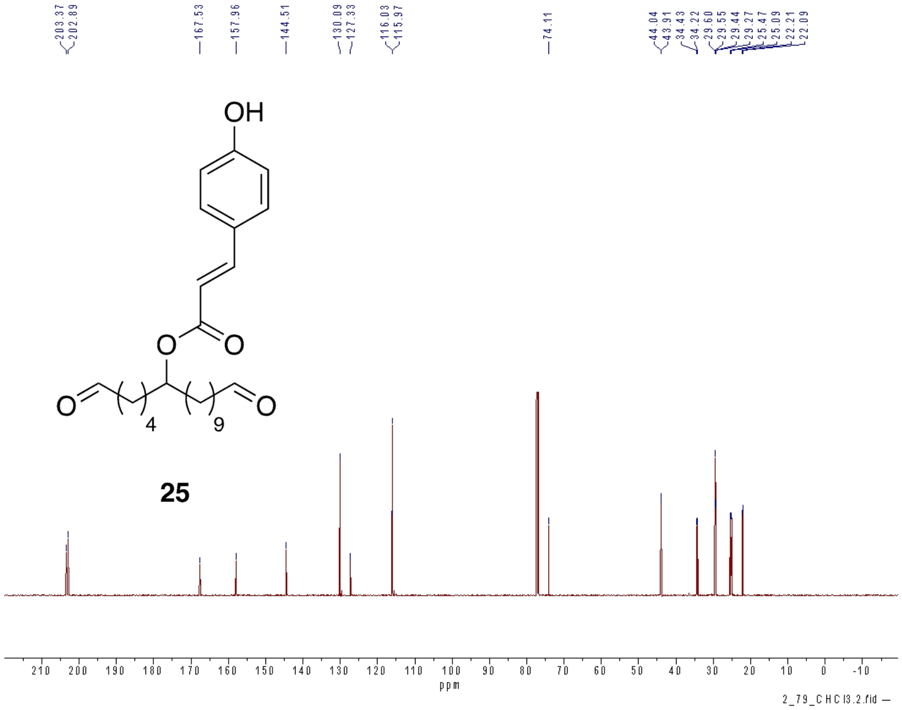

Figure S10. The ^{13}C NMR spectrum of compound **25**

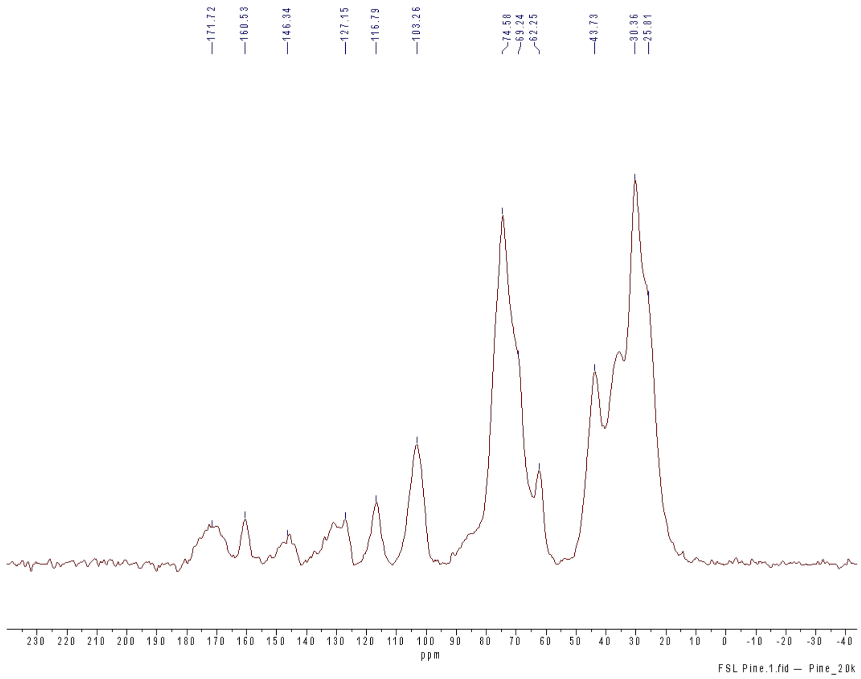

Figure S11. The ^{13}C MAS ssNMR spectrum of authentic *P. rigida* sporopollenin (*9*)

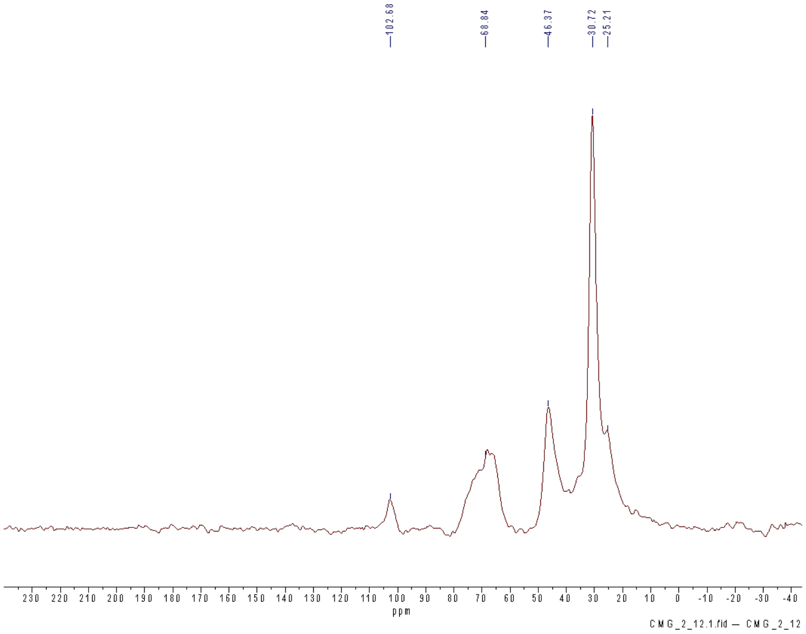

Figure S12. The ^{13}C MAS ssNMR spectrum of simplified sporopollenin analogue **15**

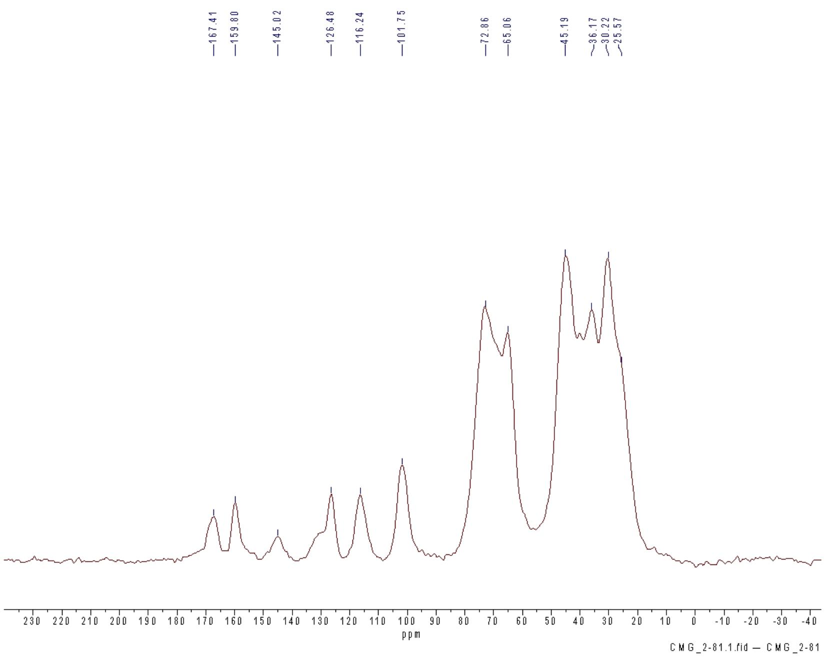

Figure S13. The ^{13}C MAS ssNMR spectrum of synthetic linker sporopollenin analogue **26**

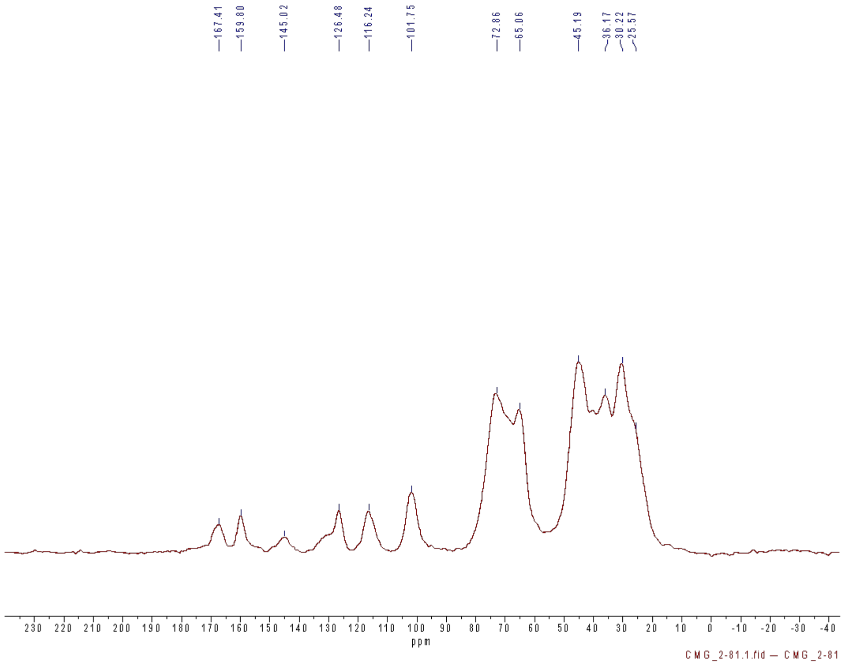

Figure S14. Overlaid ^{13}C MAS ssNMR spectra of authentic *P. rigida* sporopollenin (blue) (*9*), simplified sporopollenin analogue **15** (green), and synthetic linker sporopollenin analogue **26** (red)

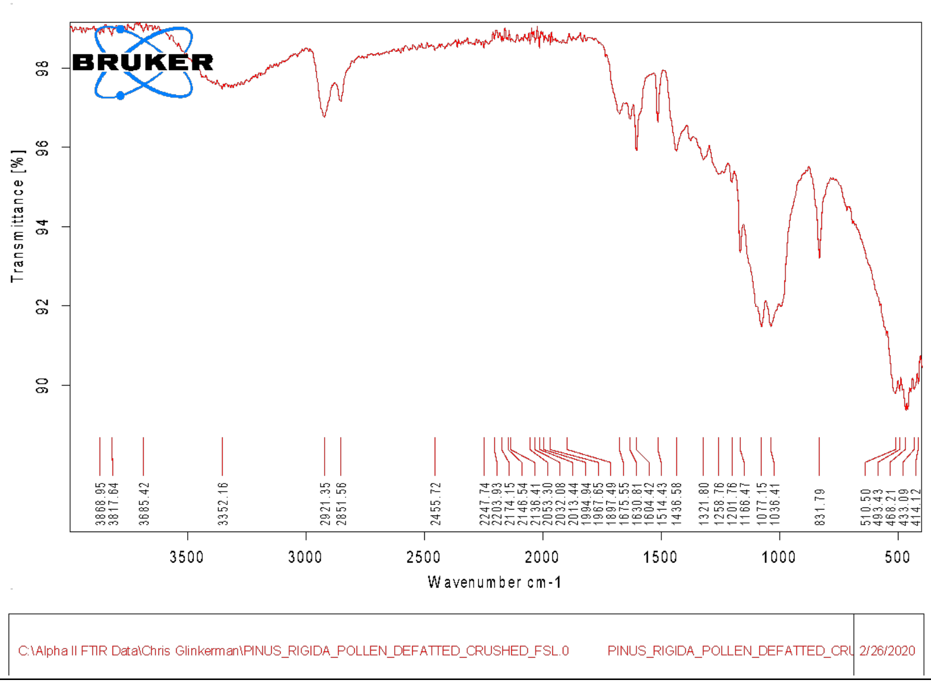

Figure S15. The IR spectrum of authentic *P. rigida* sporopollenin

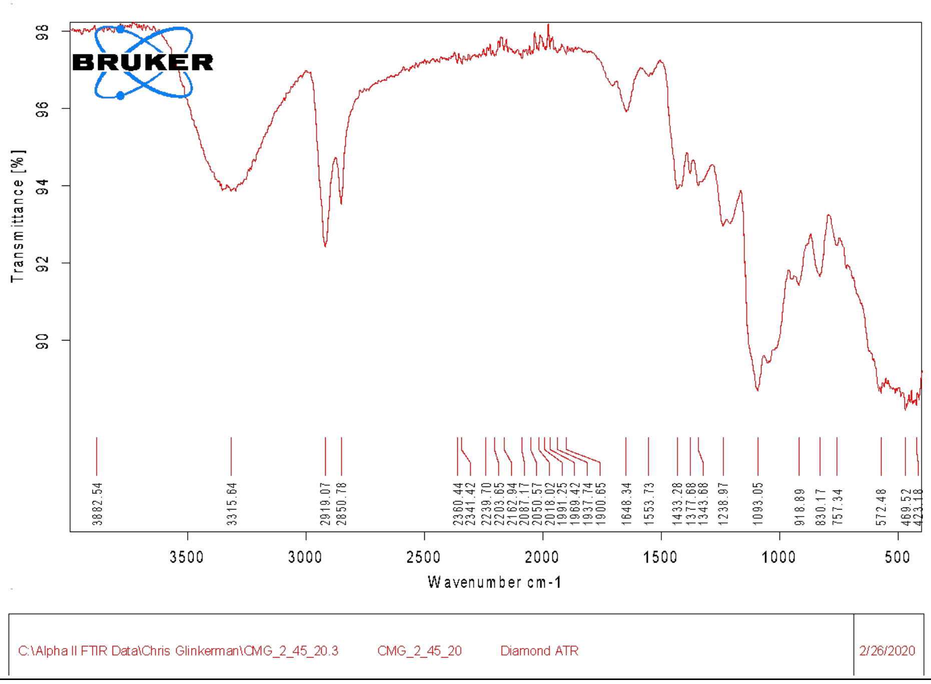

Figure S16. The IR spectrum of simplified sporopollenin analogue **15**

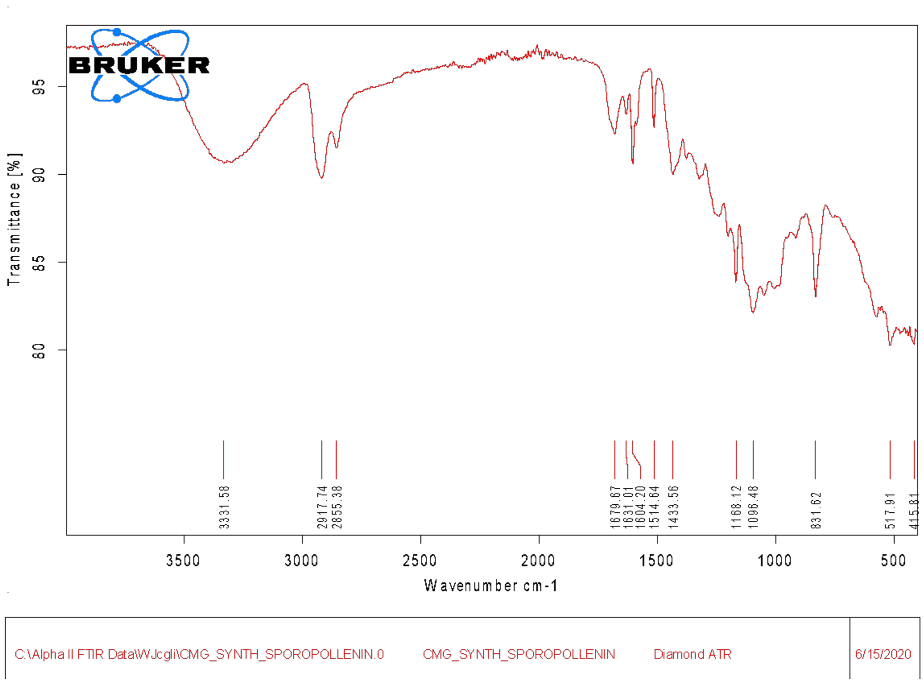

Figure S17. The IR spectrum of synthetic linker sporopollenin analogue **26**

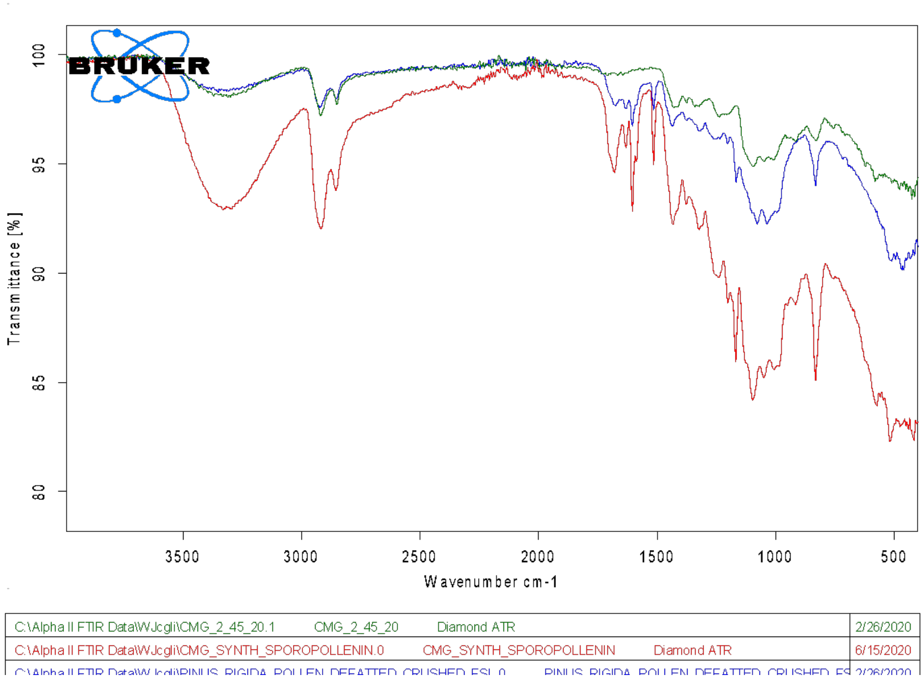

Figure S18. Overlaid IR spectra of authentic *P. rigida* sporopollenin (blue), simplified sporopollenin analogue **15** (green), and synthetic linker sporopollenin analogue **26** (red)

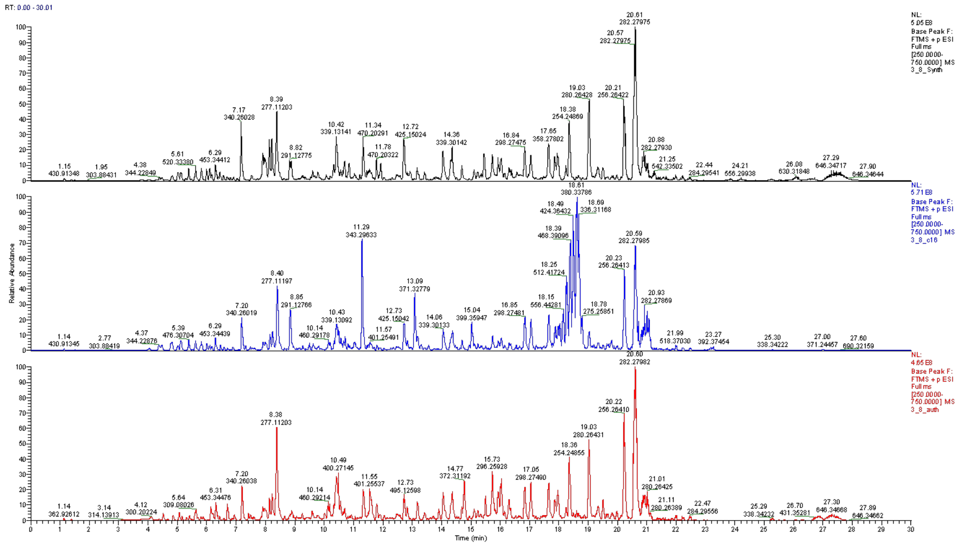

Figure S19. The base peak chromatograms of thioacidolyzed authentic *P. rigida* sporopollenin (red), simplified sporopollenin analogue **15** (blue), and synthetic linker sporopollenin analogue **26** (black)

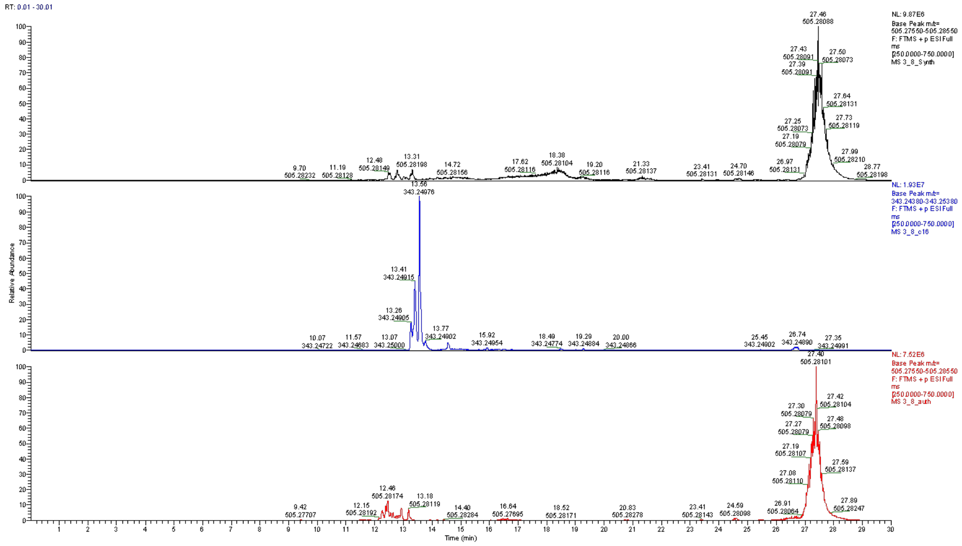

Figure S20. The extracted-ion chromatograms of thioacidolyzed authentic *P. rigida* sporopollenin (red), simplified sporopollenin analogue **15** (blue), and synthetic linker sporopollenin analogue **26** (black)

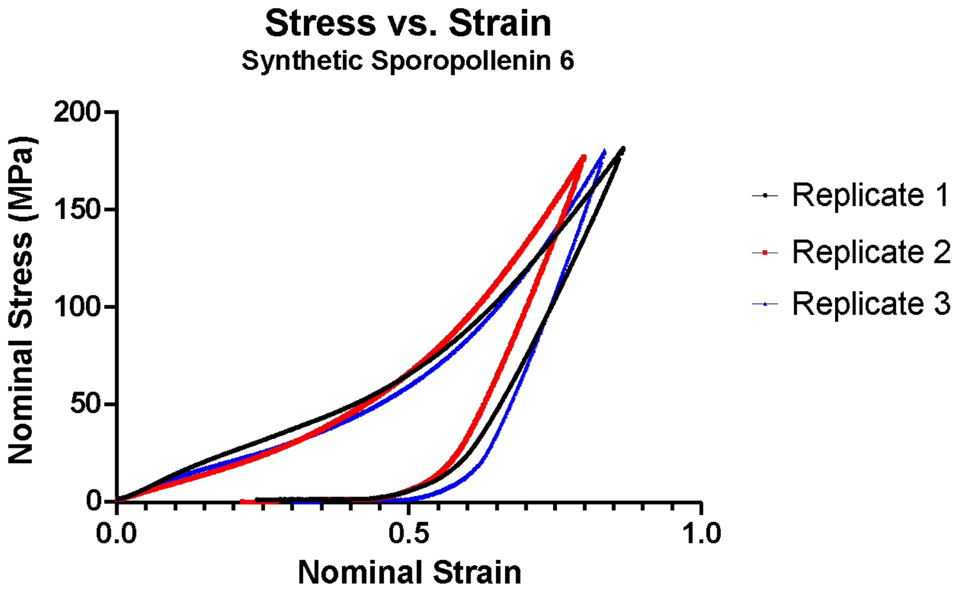

Figure S21. Compressive stress-strain curve for synthetic sporopollenin analogue **6**

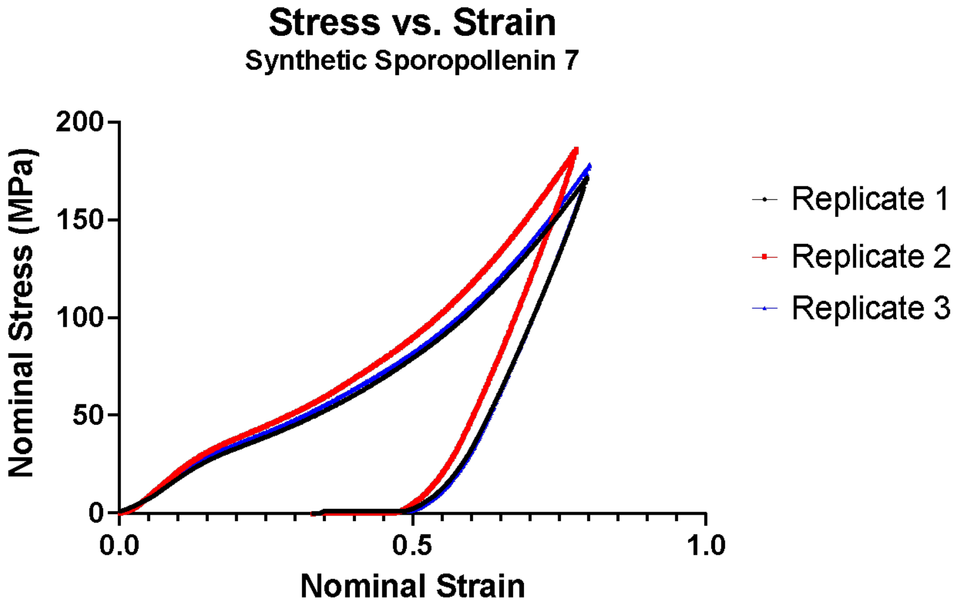

Figure S22. Compressive stress-strain curve for synthetic sporopollenin analogue **7**

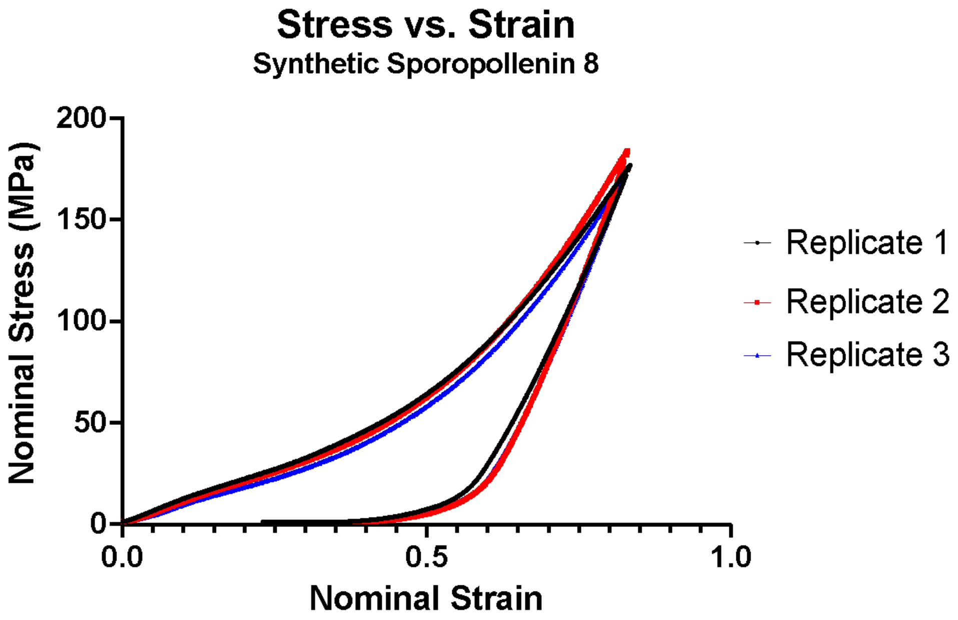

Figure S23. Compressive stress-strain curve for synthetic sporopollenin analogue **8**

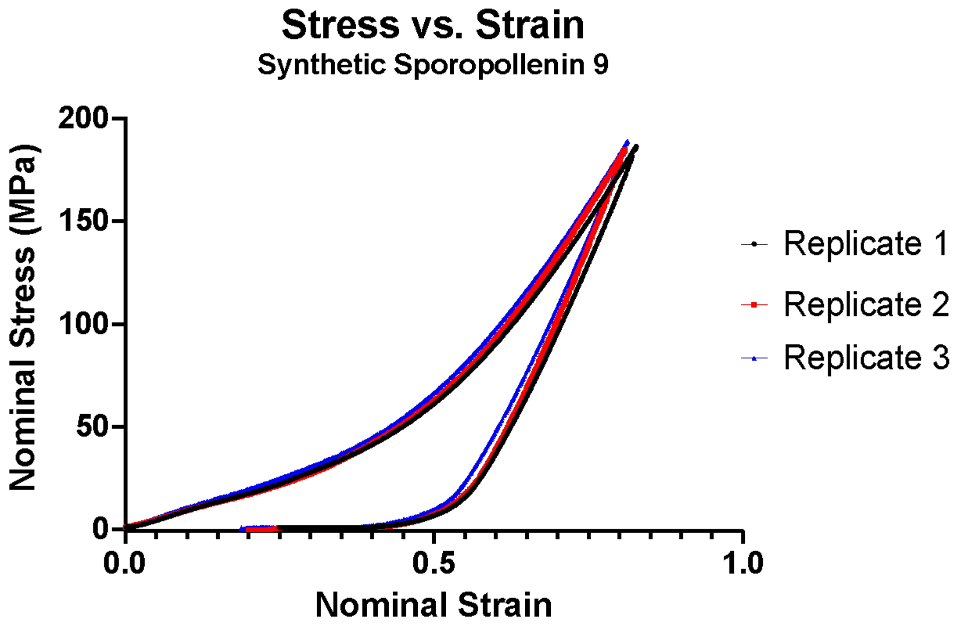

Figure S24. Compressive stress-strain curve for synthetic sporopollenin analogue **9**

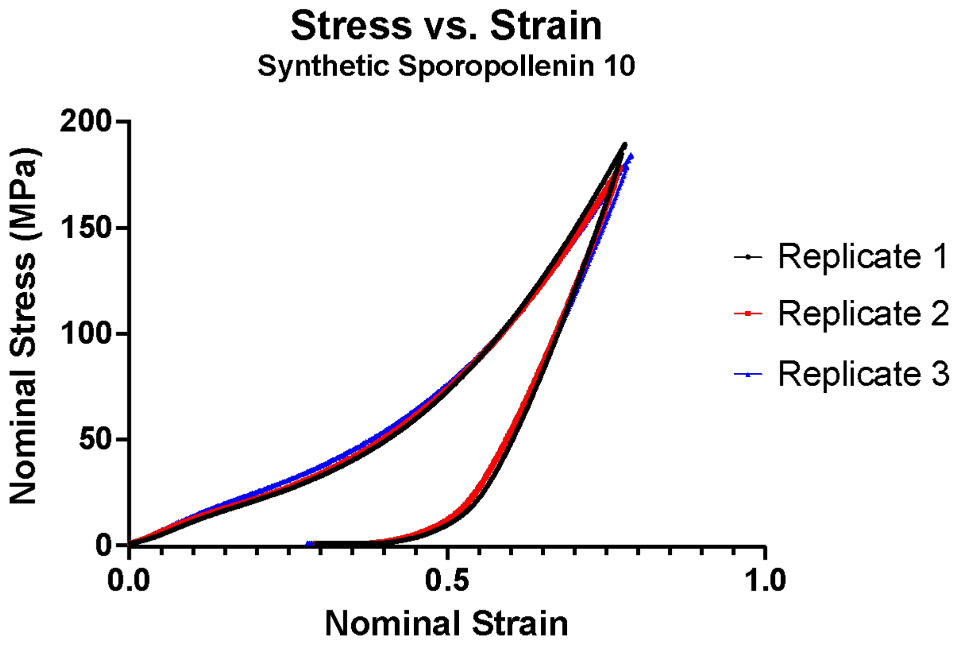

Figure S25. Compressive stress-strain curve for synthetic sporopollenin analogue **10**

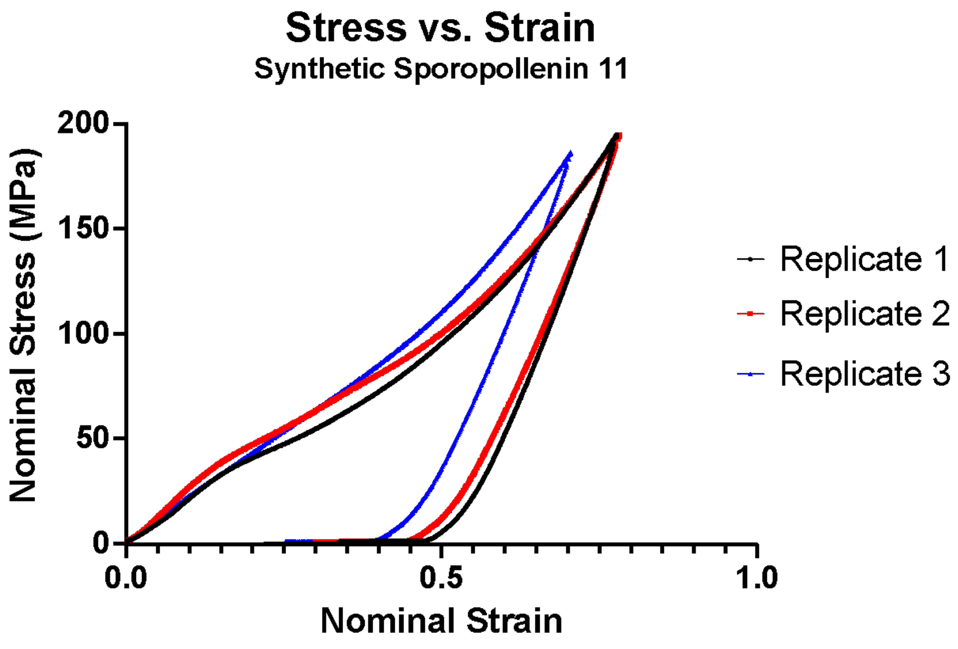

Figure S26. Compressive stress-strain curve for synthetic sporopollenin analogue **11**

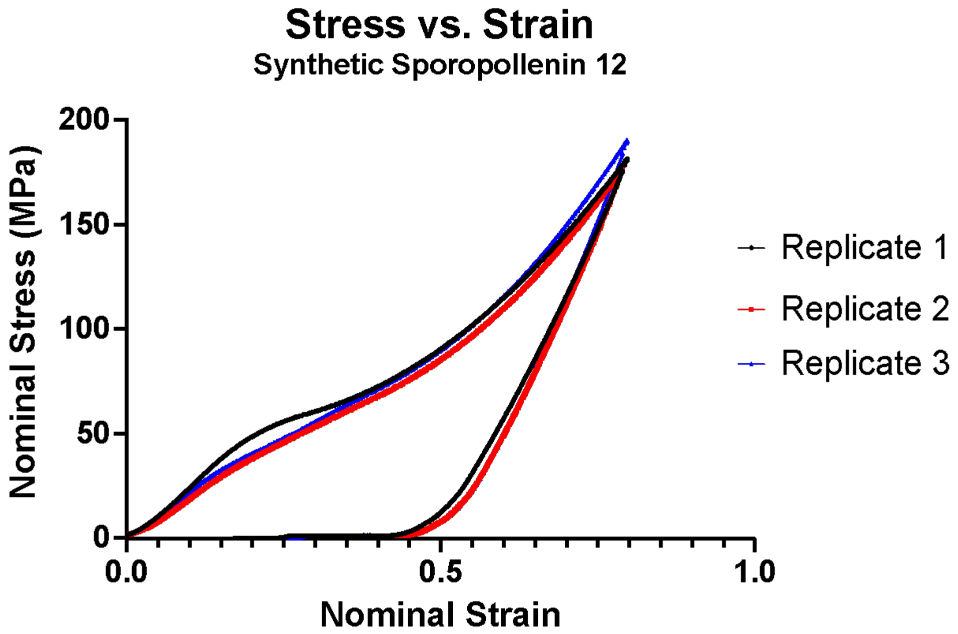

Figure S27. Compressive stress-strain curve for synthetic sporopollenin analogue **12**

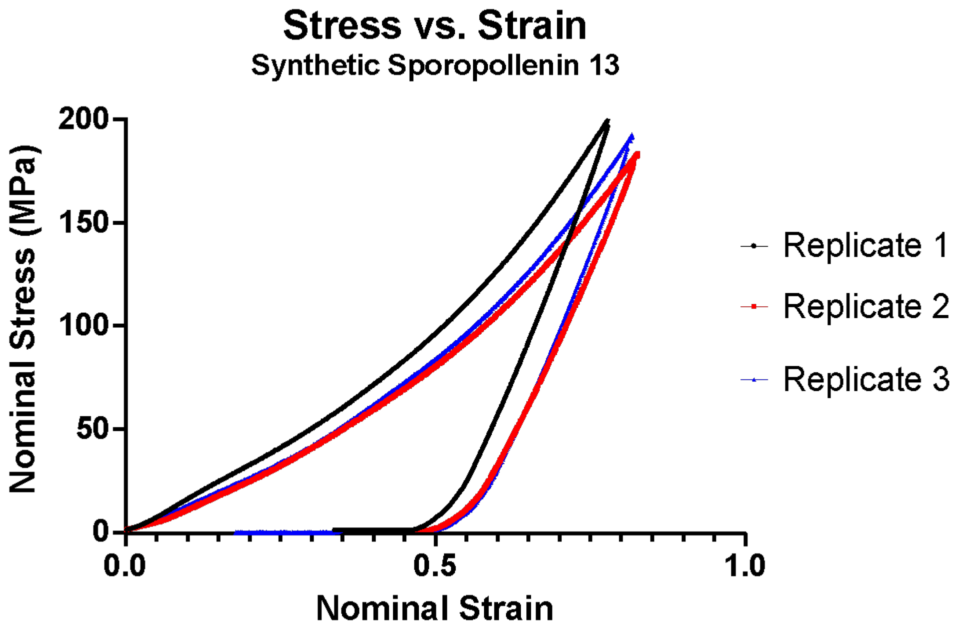

Figure S28. Compressive stress-strain curve for synthetic sporopollenin analogue **13**

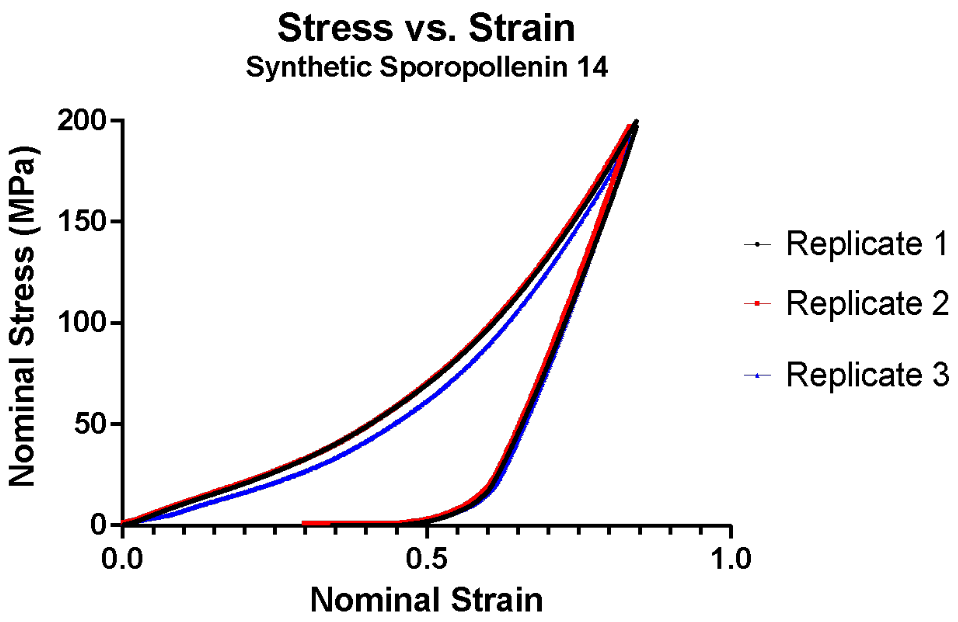

Figure S29. Compressive stress-strain curve for synthetic sporopollenin analogue **14**

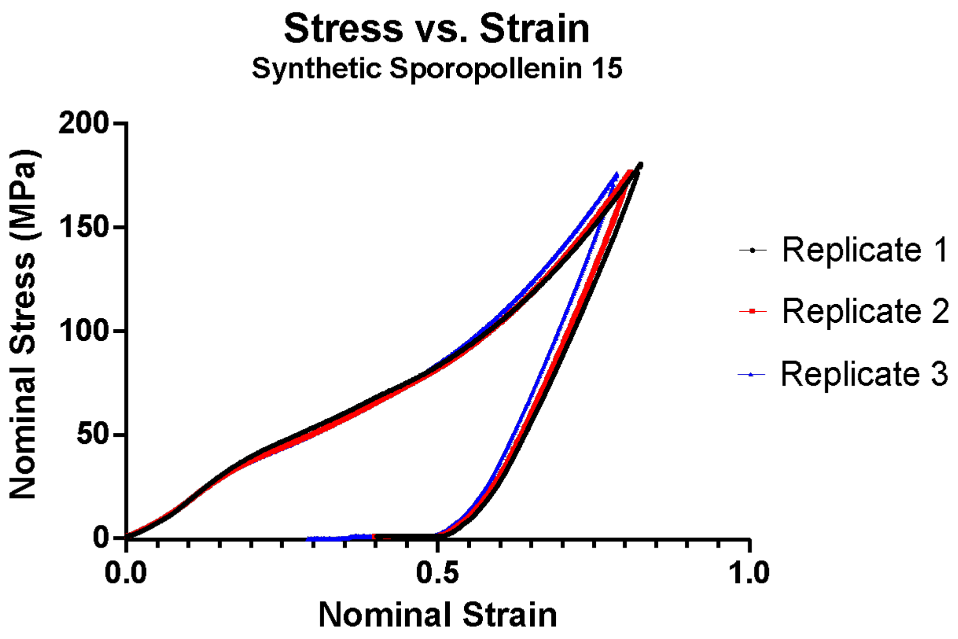

Figure S30. Compressive stress-strain curve for synthetic sporopollenin analogue **15**

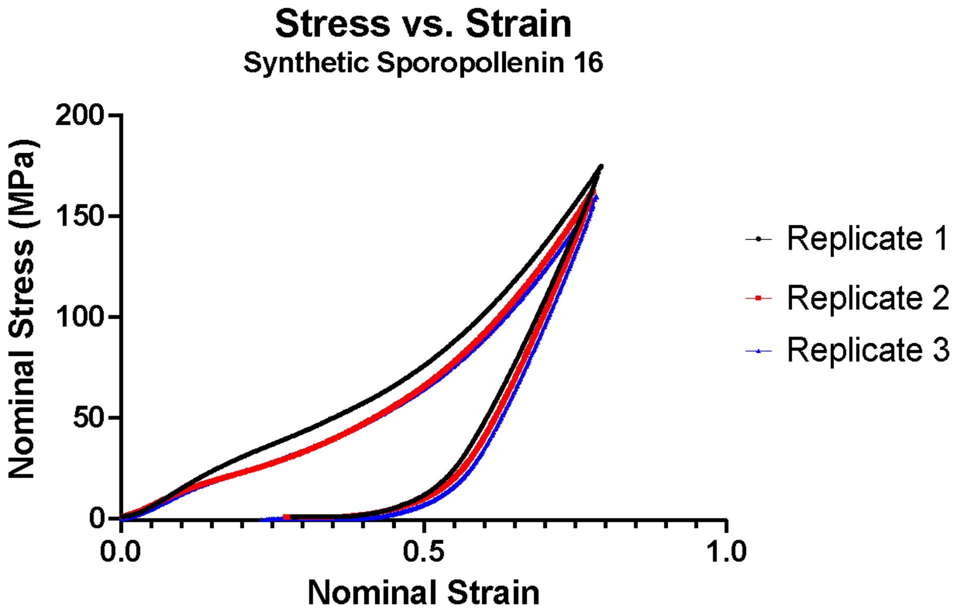

Figure S31. Compressive stress-strain curve for synthetic sporopollenin analogue **16**

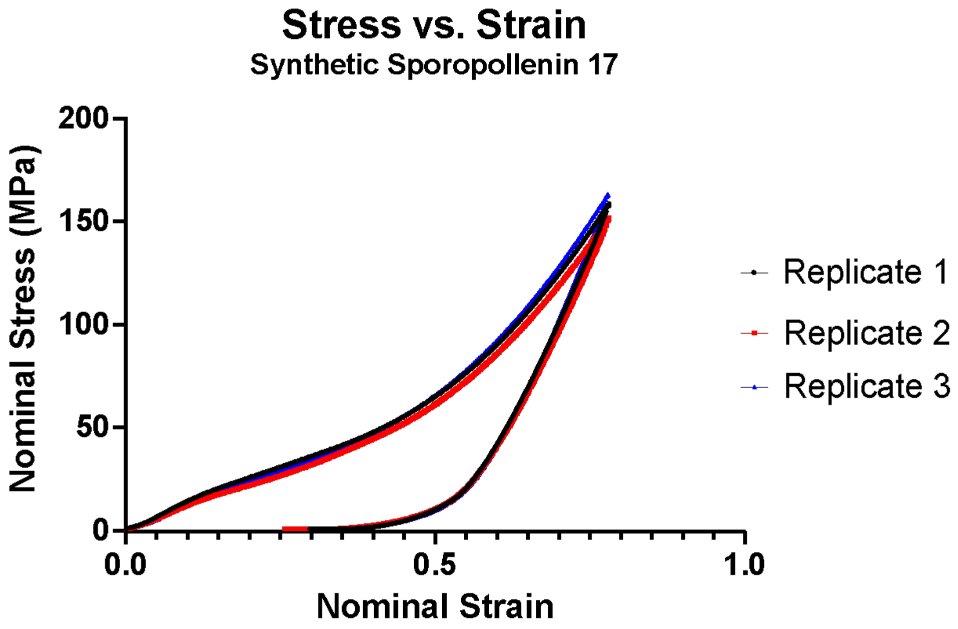

Figure S32. Compressive stress-strain curve for synthetic sporopollenin analogue **17**

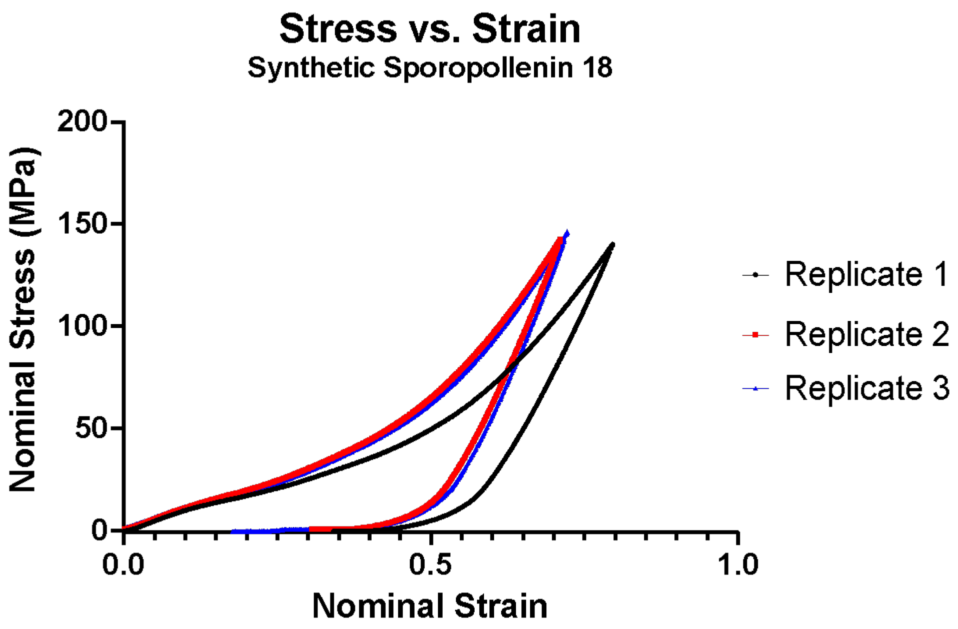

Figure S33. Compressive stress-strain curve for synthetic sporopollenin analogue **18**

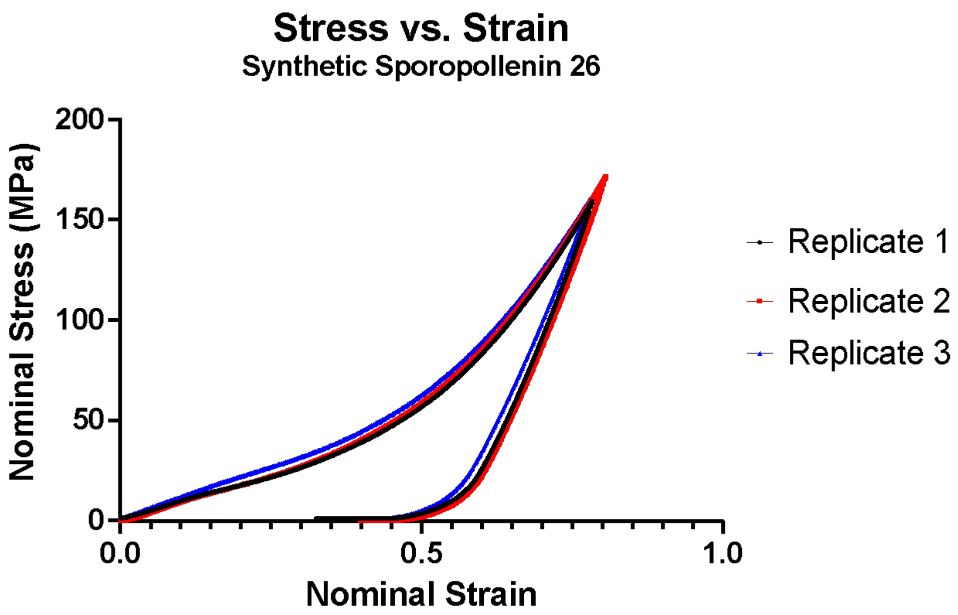

Figure S34. Compressive stress-strain curve for synthetic sporopollenin analogue **26**

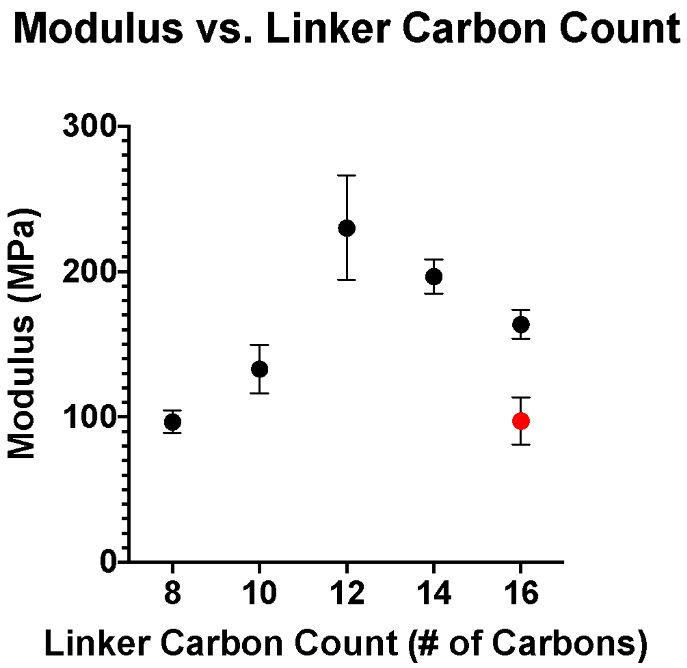

Figure S35. Relationship between compressive modulus and linker carbon count in simplified (black) and substituted (red) synthetic linker sporopollenin analogues (error bars = standard deviation)

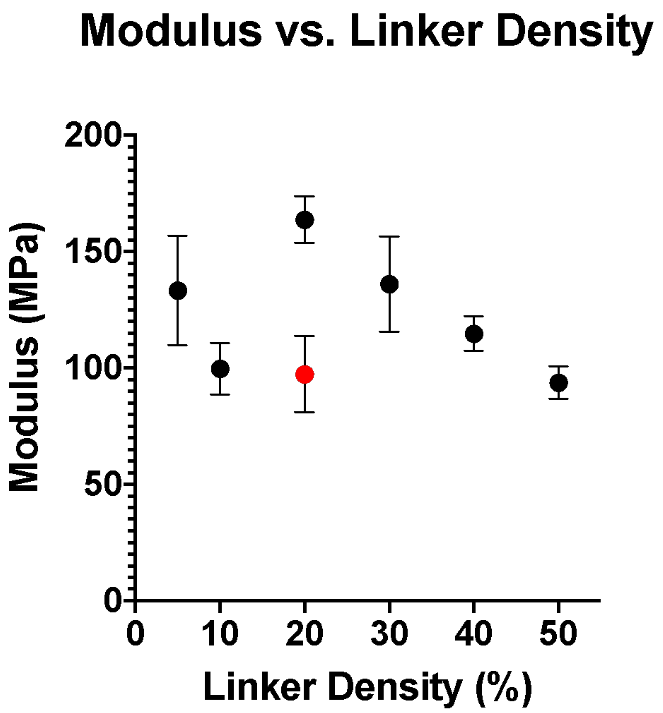

Figure S36. Relationship between compressive modulus and linker density in simplified (black) and substituted (red) synthetic linker sporopollenin analogues (error bars = standard deviation)

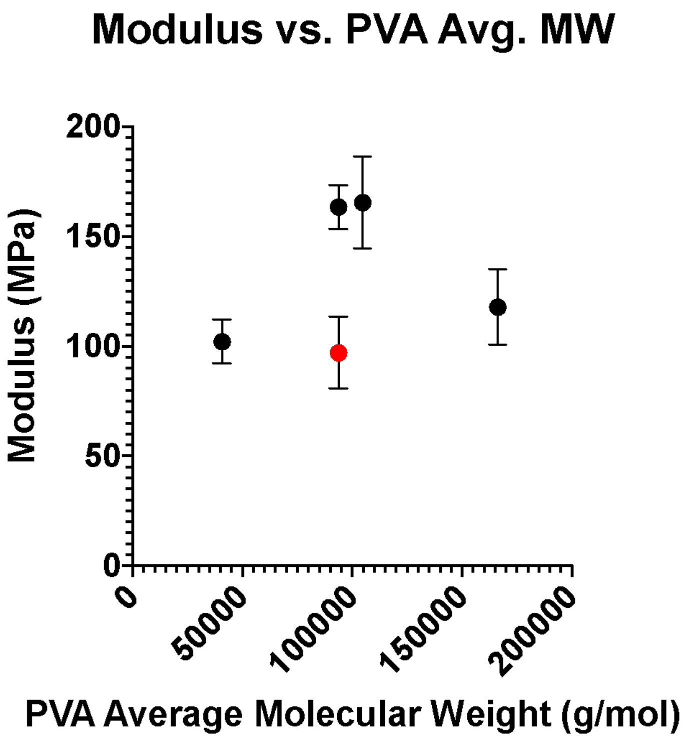

Figure S37. Relationship between compressive modulus and PVA MW_{avg} in simplified (black) and substituted (red) synthetic linker sporopollenin analogues (error bars = standard deviation)

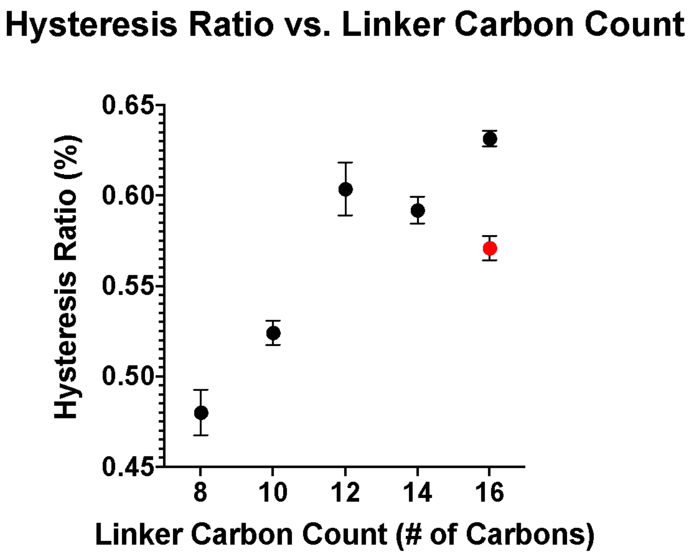

Figure S38. Relationship between compressive hysteresis ratio and linker carbon count in simplified (black) and substituted (red) synthetic linker sporopollenin analogues (error bars = standard deviation)

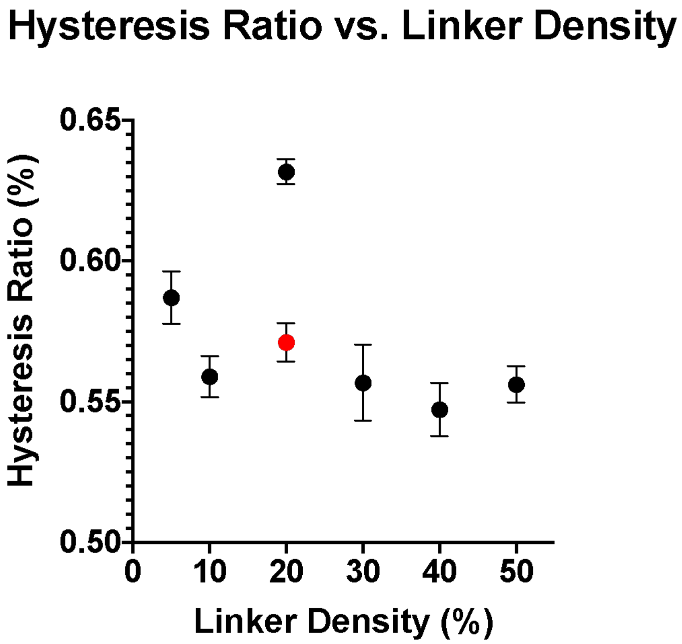

Figure S39. Relationship between compressive hysteresis ratio and linker density in simplified (black) and substituted (red) synthetic linker sporopollenin analogues (error bars = standard deviation)

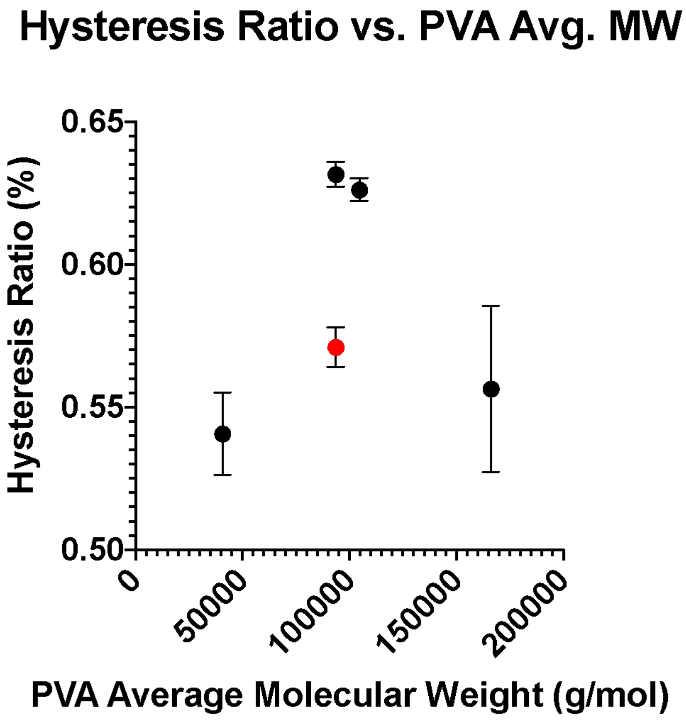

Figure S40. Relationship between compressive hysteresis ratio and PVA MW_{avg} in simplified (black) and substituted (red) synthetic linker sporopollenin analogues (error bars = standard deviation)

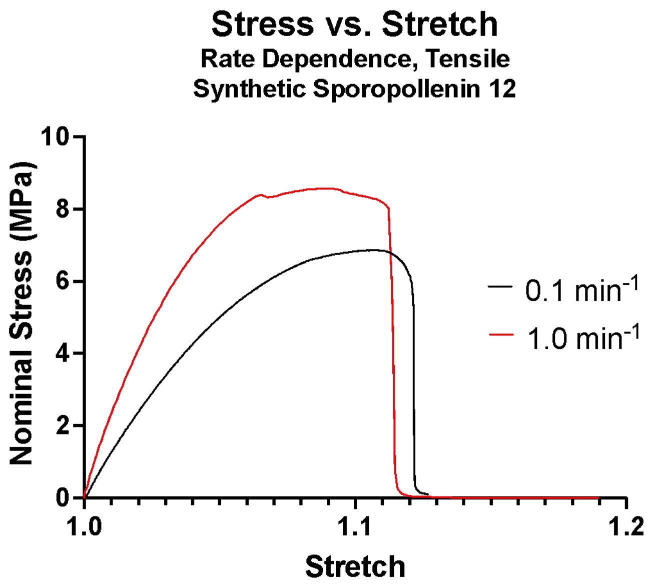

Figure S41. Tensile stress-strain curve for synthetic sporopollenin analogue **12** at 1.0 (red) and 0.1 min^{-1} (black) loading rates

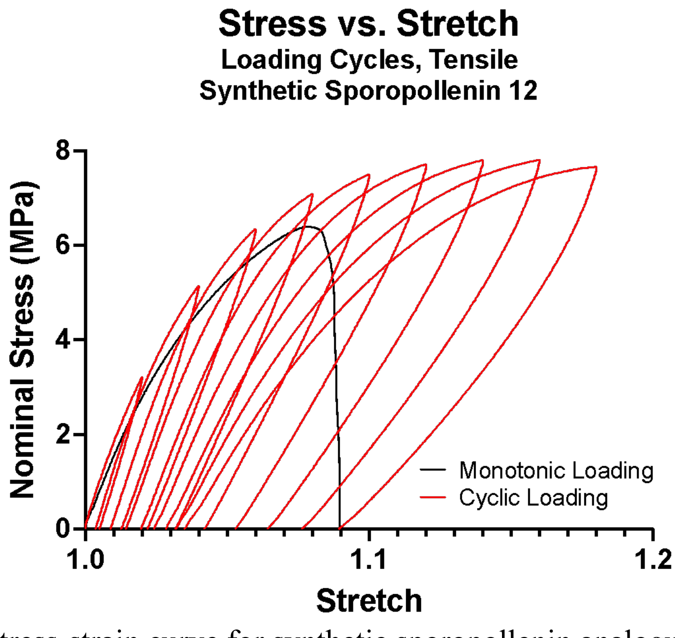

Figure S42. Tensile stress-strain curve for synthetic sporopollenin analogue **12** with cyclic (red) and monotonic (black) loading

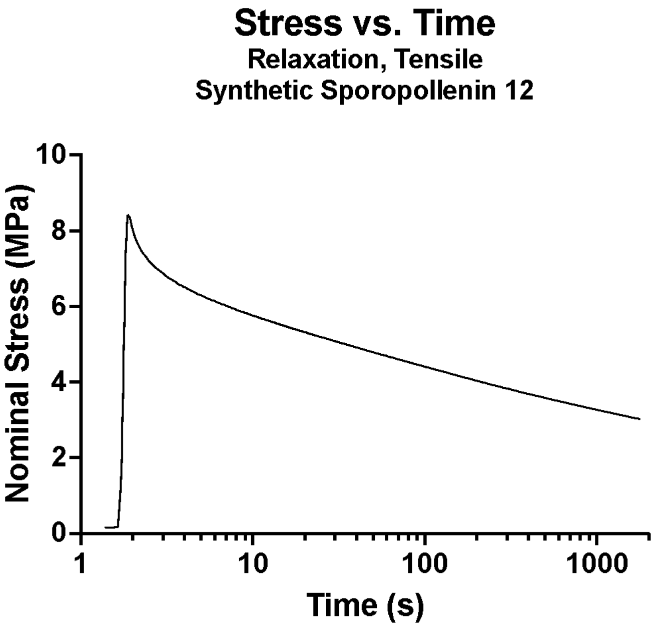

Figure S43. Tensile stress-time relaxation curve for synthetic sporopollenin analogue **12**

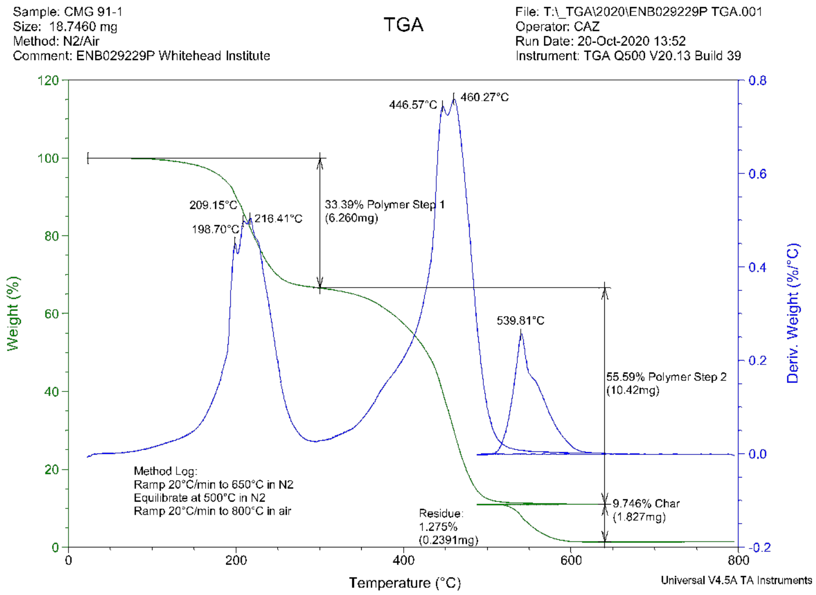

Figure S44. The TGA thermogram for synthetic sporopollenin analogue **6**

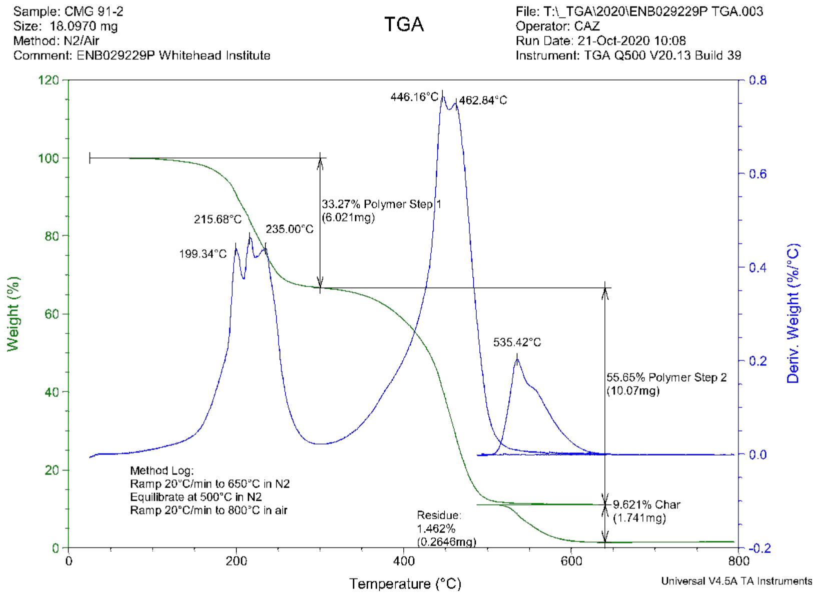

Figure S45. The TGA thermogram for synthetic sporopollenin analogue **7**

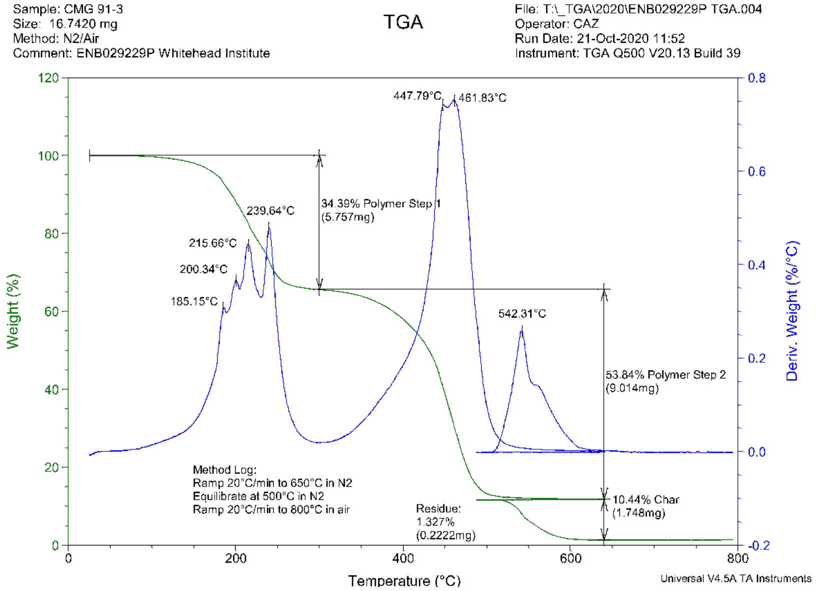

Figure S46. The TGA thermogram for synthetic sporopollenin analogue **8**

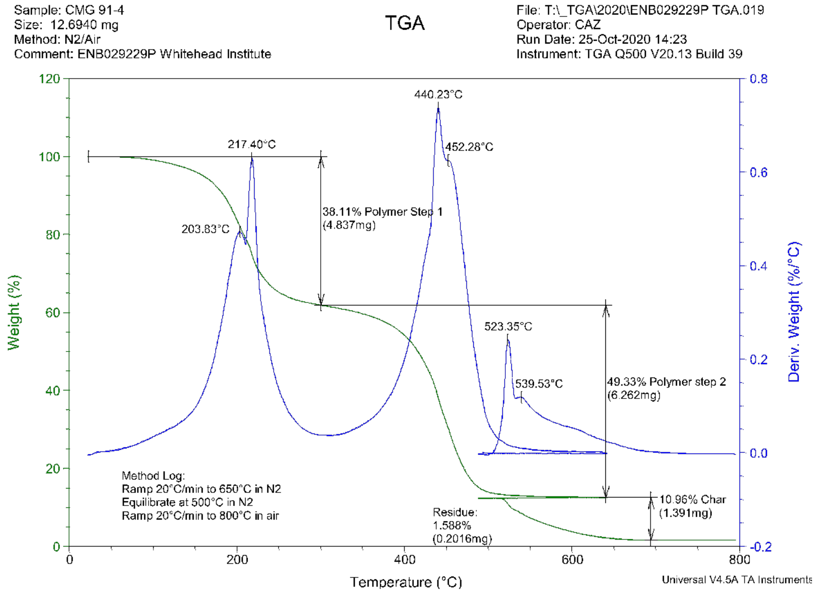

Figure S47. The TGA thermogram for synthetic sporopollenin analogue **9**

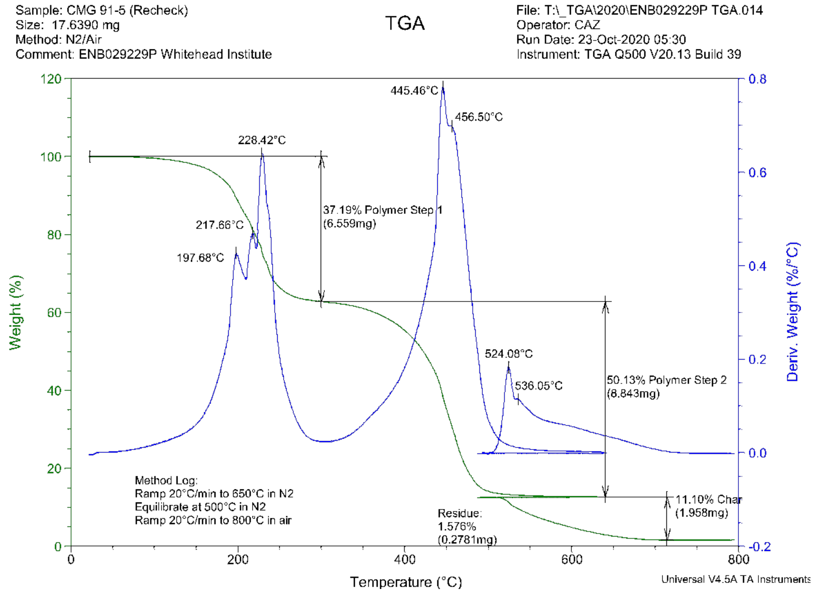

Figure S48. The TGA thermogram for synthetic sporopollenin analogue **10**

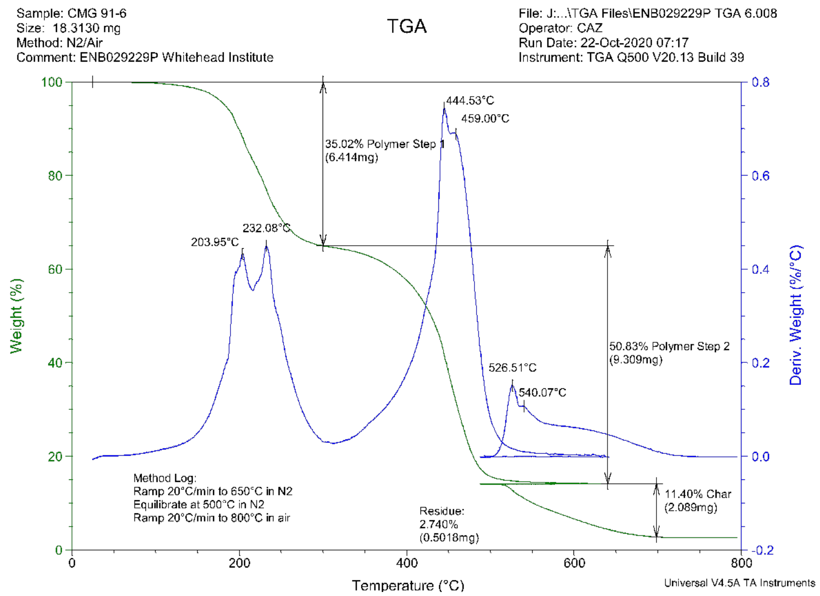

Figure S49. The TGA thermogram for synthetic sporopollenin analogue **11**

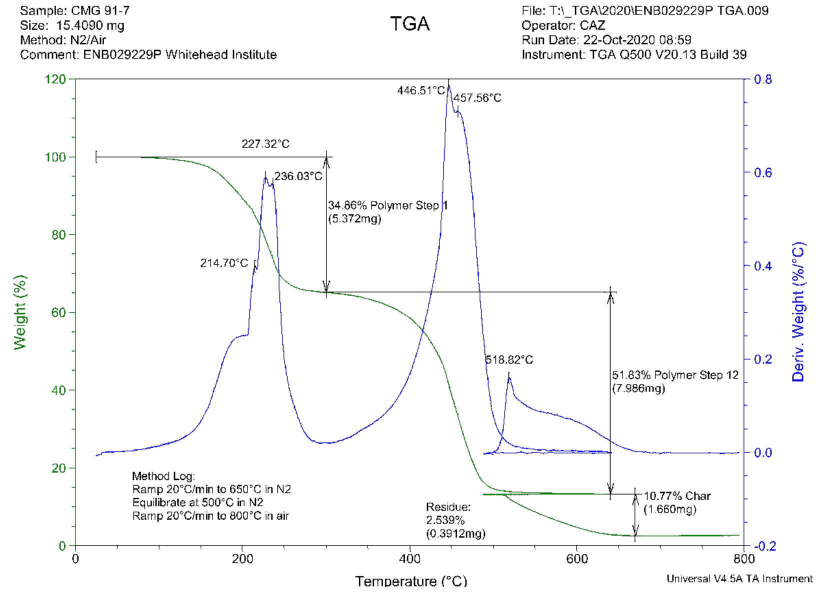

Figure S50. The TGA thermogram for synthetic sporopollenin analogue **12**

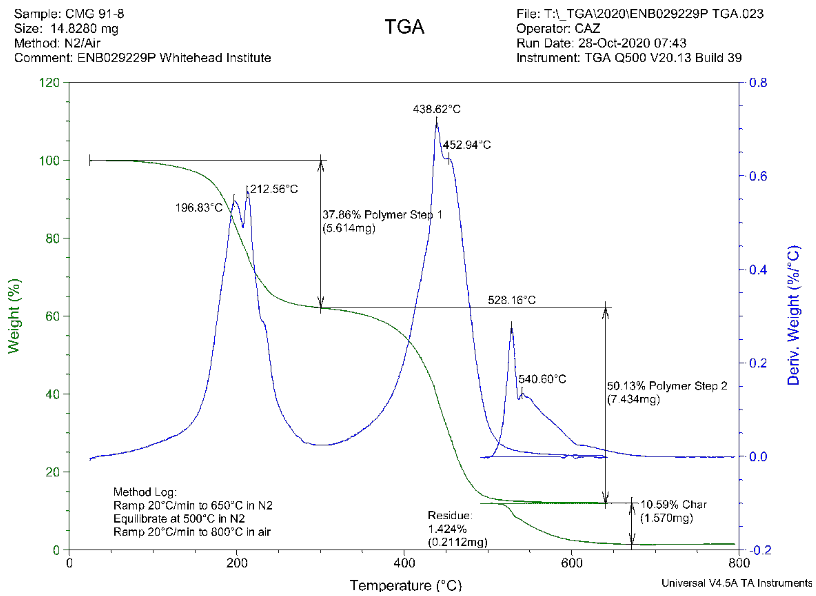

Figure S51. The TGA thermogram for synthetic sporopollenin analogue **13**

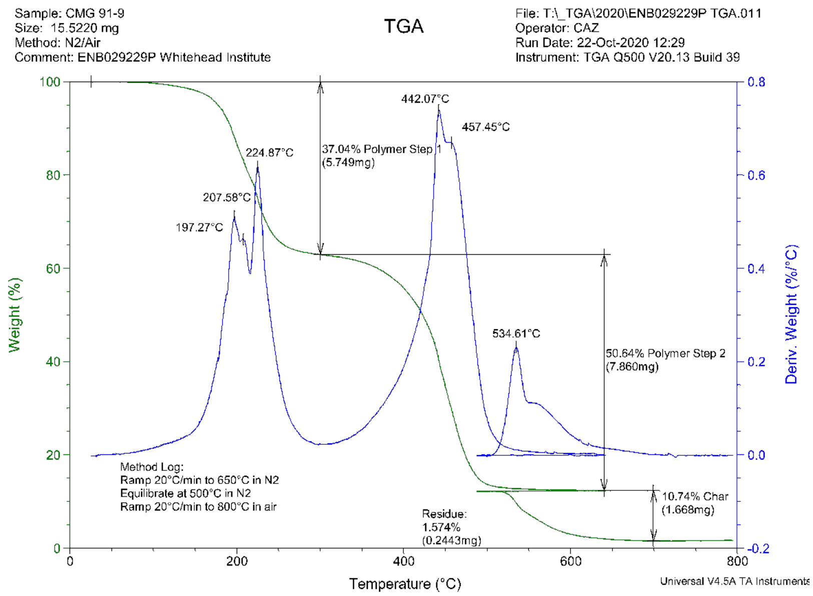

Figure S52. The TGA thermogram for synthetic sporopollenin analogue **14**

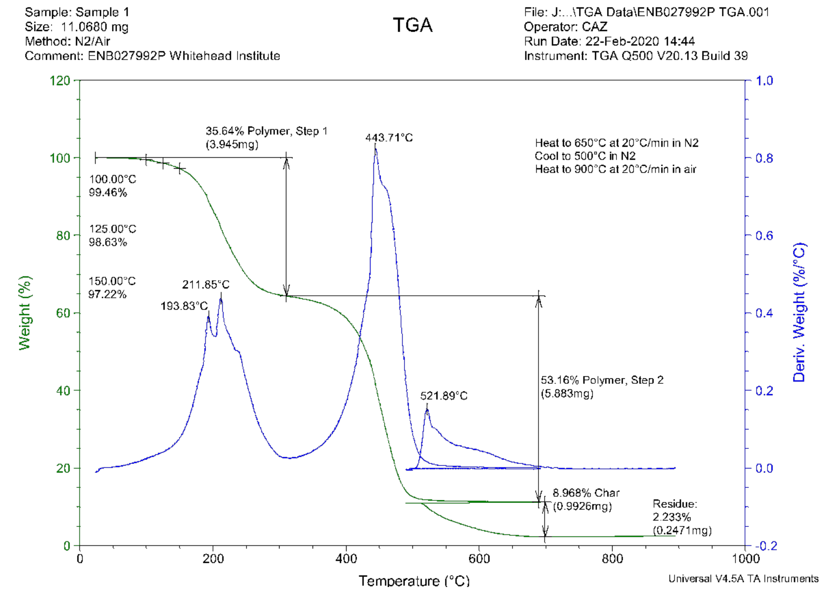

Figure S53. The TGA thermogram for synthetic sporopollenin analogue **15**

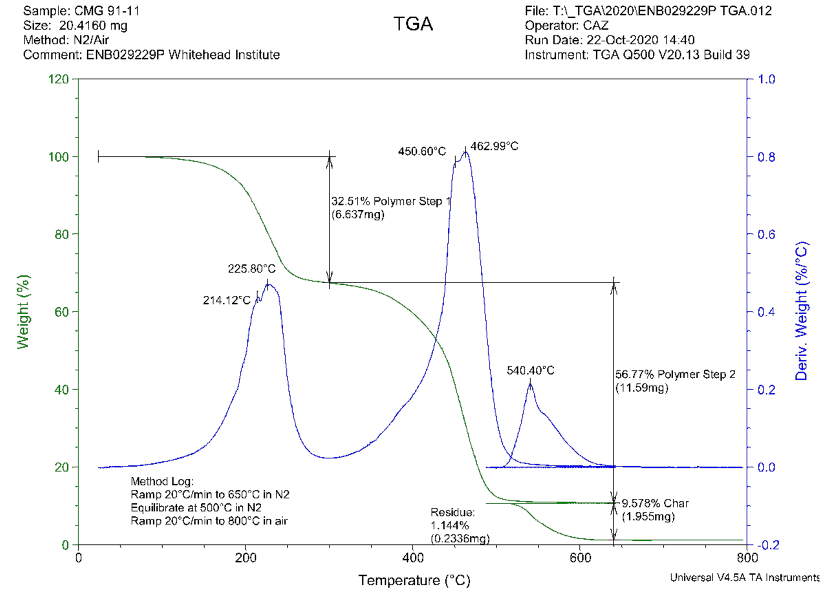

Figure S54. The TGA thermogram for synthetic sporopollenin analogue **16**

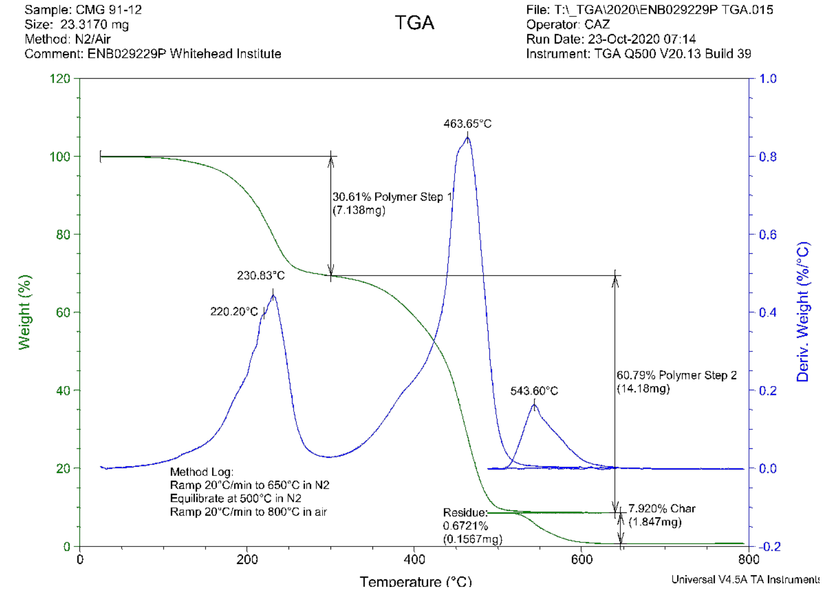

Figure S55. The TGA thermogram for synthetic sporopollenin analogue **17**

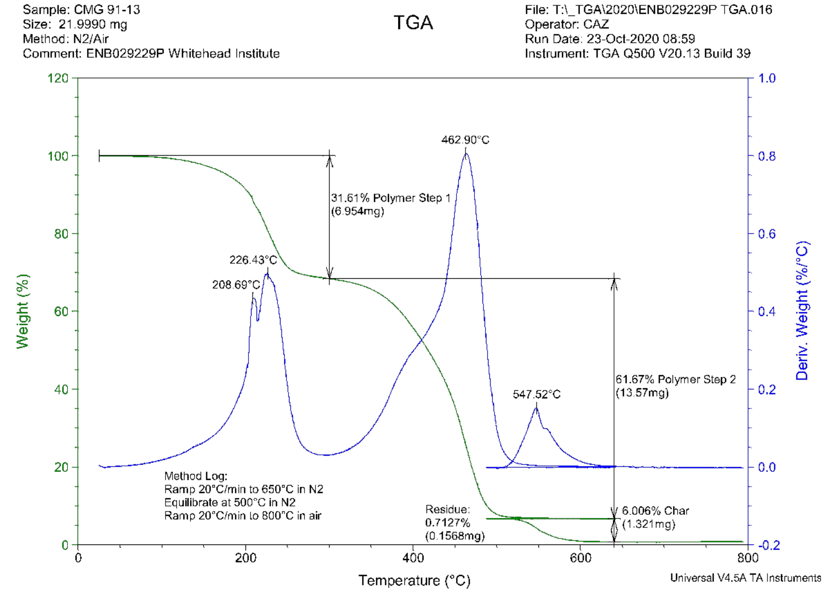

Figure S56. The TGA thermogram for synthetic sporopollenin analogue **18**

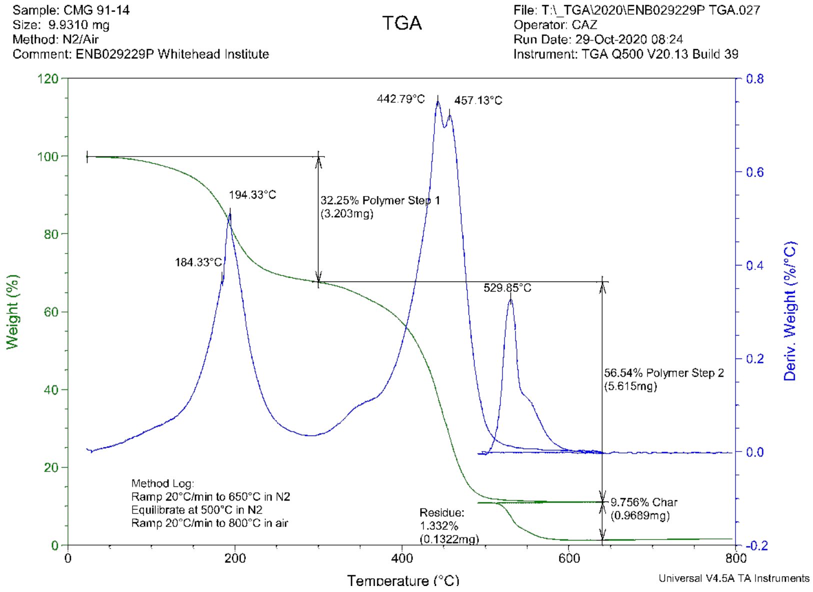

Figure S57. The TGA thermogram for synthetic sporopollenin analogue **26**

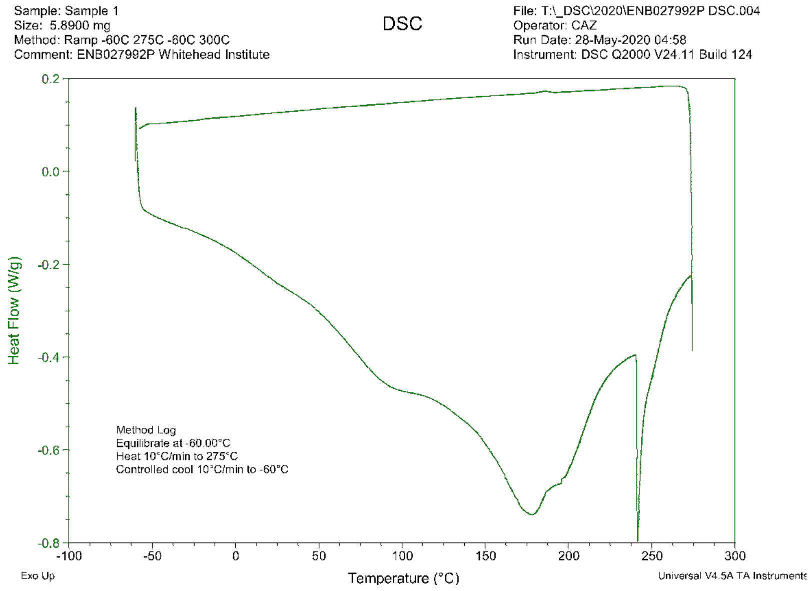

Figure S58. The DSC thermogram for synthetic sporopollenin analogue **15**, first replicate

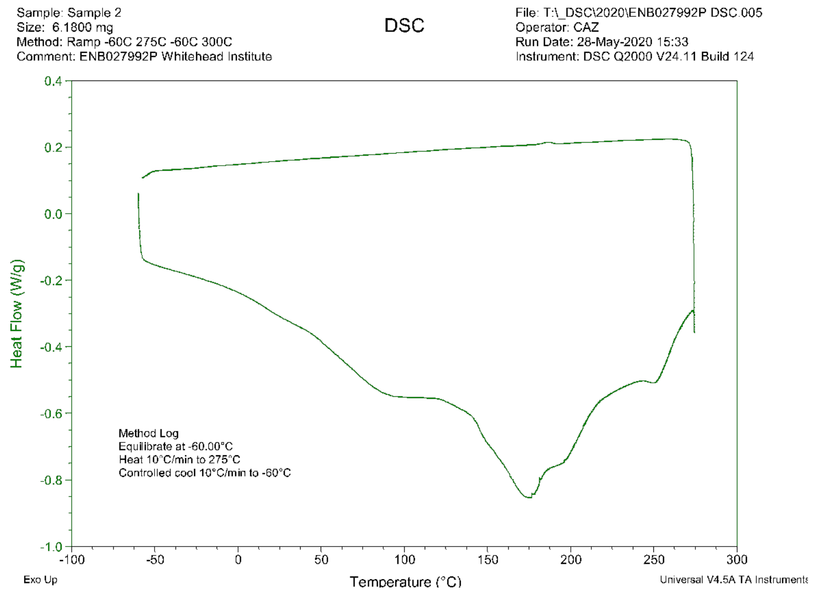

Figure S59. The DSC thermogram for synthetic sporopollenin analogue **15**, second replicate

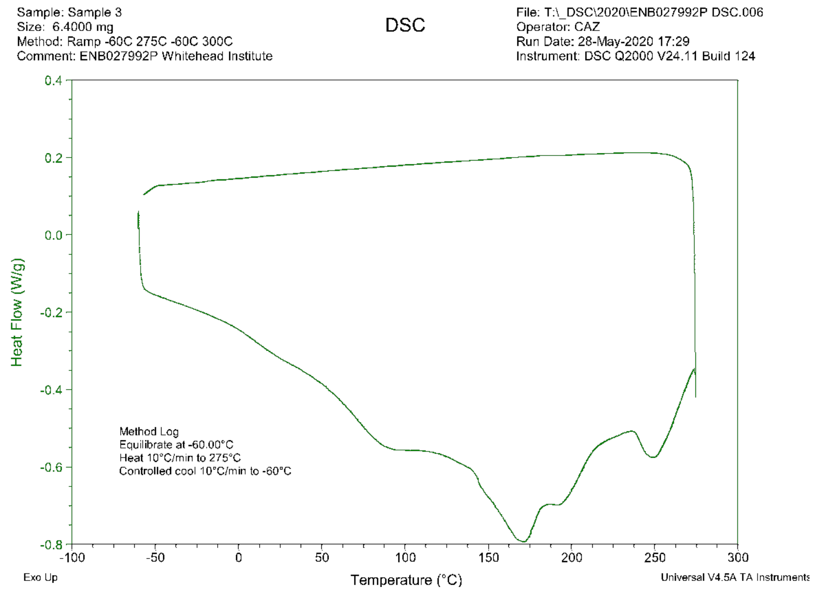

Figure S60. The DSC thermogram for synthetic sporopollenin analogue **15**, third replicate

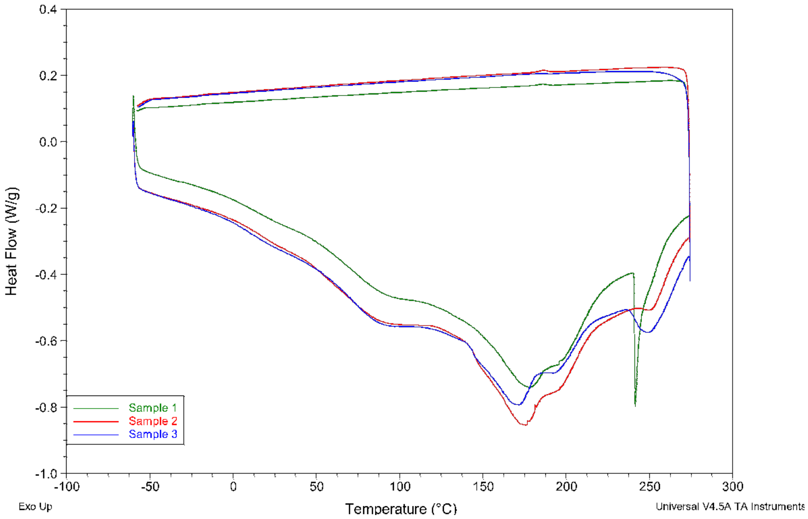

Figure S61. The DSC thermogram for synthetic sporopollenin analogue **15**, all replicates

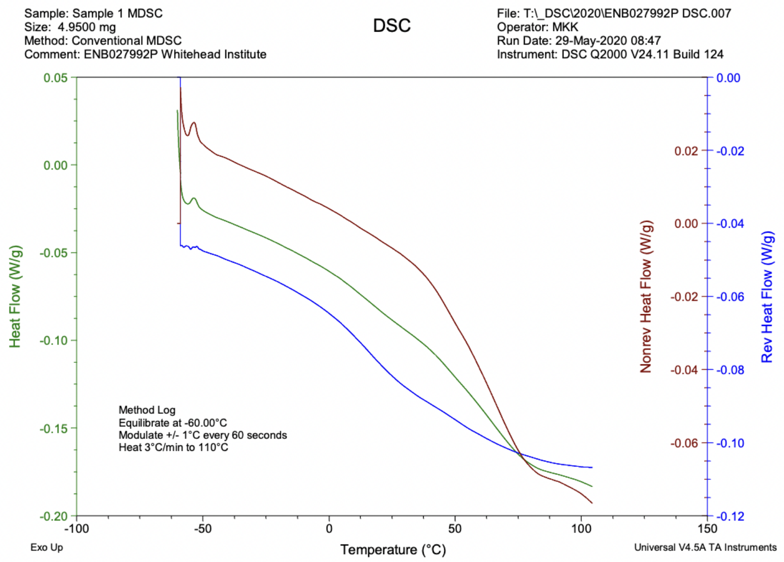

Figure S62. The modulated DSC thermogram for synthetic sporopollenin analogue **15**
