# Supplementary material for: Sporopollenin-inspired design and synthesis of robust polymeric materials
Source: Commun Chem. 2022 Sep 12;5:110. doi: 10.1038/s42004-022-00729-w (PMC9814627; doi:10.1038/s42004-022-00729-w)
Supplement: Supplementary file 1 — Weng_PR File [file 42004_2022_729_MOESM1_ESM.pdf]

nature portfolio

Peer Review File

Sporopollenin-inspired design and synthesis of robust polymeric materials

Open Access This file is licensed under a Creative Commons Attribution 4.0 International License, which permits use, sharing, adaptation, distribution and reproduction in any medium or format, as long as you give appropriate credit to the original author(s) and the source, provide a link to the Creative Commons license, and indicate if changes were made. In the cases where the authors are anonymous, such as is the case for the reports of anonymous peer reviewers, author attribution should be to 'Anonymous Referee' followed by a clear attribution to the source work. The images or other third party material in this file are included in the article's Creative Commons license, unless indicated otherwise in a credit line to the material. If material is not included in the article's Creative Commons license and your intended use is not permitted by statutory regulation or exceeds the permitted use, you will need to obtain permission directly from the copyright holder. To view a copy of this license, visit http://creativecommons.org/licenses/by/4.0/.

Reviewers' comments:

Reviewer #1 (Remarks to the Author):

The authors demonstrated in this manuscript that PVA-based cross-linked polymers were synthesized as novel robust materials inspired by sporopollenin. The synthetic strategy to mimic the plant-derived stiff biopolymer is rationally designed to achieve a biomimetic chemical structure resembling the sporopollenin. The structural analysis on the synthesized polymers seems to scientifically sound well, but I have uncertain points in the material evaluation. The detailed comments are listed below:

1) The plausible chemical structure of sporopollenin, which was revealed by the authors in previous work, contains fatty acid and alpha-pyrone moieties at the terminal of polyketide backbone. According to the chemical composition in the previous work, I think these components cannot be ignored for the design of sporopollenin-mimetic polymers. Why did the authors omit such components for this work?

2) The authors claimed that the combination of ester and acetal linkages in the cross-linked structure of sporopollenin is quite important for the stability of this biomaterial in the previous study. Why not the authors try to incorporate ester linkages (probably based on fatty acid moiety in sporopollenin)?

3) In TGA of the synthetic sporopollenin analogues, the authors concluded that the weight loss at around 200 oC is attributed to residual solvent DMSO. However, the weight loss at 200 oC reaches 35% for all the samples, which means the materials are organogels rather than cross-linked polymers. Is there possibility that additional thermal condensation occurred during TGA process? I recommend the authors to analyze the exact amount of residual solvent by other methods (NMR, elementary analysis, etc). Such the large amount of residual solvent should also affect the mechanical properties of the polymers.

4) In Figure 4D, the legends for color bars are missing.

5) For the mechanical properties (Shore hardness, compression study), a control sample (namely, native sporopollenin) should be required for comparative analysis.

6) The modulus of cross-linked polymers was strongly related to the molecular weight of inter-crosslinking points. Therefore, generally the more cross-linked the polymer is, the higher moduli of the polymers become. As the cross-linking density increased in Table S5 (polymers 13-18), the observed moduli tend to decrease. The authors need clearer explanation about this.

7) The synthesized polymer 26 containing coumaric acid moiety is the most similar structure to sporopollenin. Since the other analogues are based on PVA cross-linked aldehyde, which are very common polymeric materials (for example, many reports on PVA cross-linked by glutaraldehyde have been reported), I recommend the authors to emphasize the specific feature of the synthesized polymer 26 on the discussion for material properties.

8) Comment: I am just curious about the role of coumaric acid moiety. Cinnamic acid analogues generally dimerize under light irradiation, making robust cross-linking if they are incorporated in polymer structures. Do the authors think that such the dimerization of coumaric acids can take place in the synthesized polymers (and also in native sporopollenin)?

Reviewer #2 (Remarks to the Author):

Weng et al. describe the bioinspired synthesis of a series of sporopollenin with discrete control over linker length, linker substituent(s), degree of crosslinking, and polyvinyl alcohol backbone properties,

including average molecular weight, polydispersity, and tacticity, measured chemical, thermal, and mechanical properties of the synthetic sporopollenins. This may provide chemical tools for the sporopollenin biosynthesis, and could encourage the development and application of robust sporopollenin-inspired polymers. However, the authors should address the following concerns.

1. In Figure 1, author shows electron micrograph detailed hypothesis for the molecular structure of P. rigida sporopollenin. This could perhaps go to supplementary information as the structural model of the sporopollenin have been confirmed by authors' previous article (Nat. Plants 2019, 5, 41.).
2. The authors have obtained a series of sporopollenin-inspired polymers from chemical synthesis with different linker carbon count, linker density and PVA MW, that is basis for selecting these parameters. Why not we can see the combination of linker carbon count (8 #C), linker density (10 %) and PVA MW (104500 g/mol)?
3. Information on the number of tests is missing and needs to be given (number of specimens of the thermal characterization, number of mechanical properties, and number of swelling ratios).
4. The uniform hardness of sporopollenin-inspired polymers averaging 71 ± 5 Shore D regardless of crosslinker length, crosslinking density and PVA average molecular weight, what is the hardness of natural sporopollenin?
5. What are the key factors affecting compression performance of sporopollenin-inspired polymers? There is little difference in compression performance between the various sporopollenin analogues.
6. The authors have compared relationship between weight swelling ratio and crosslinker density. Are there any relationships between swelling ratio and linker carbon count PVA MW?
7. There are a series of three mass losses, so three corresponding to which number of sporopollenin analogue? Is there relationship between weight losses and synthetic?
8. It is necessary to compare the performance between natural and synthetic sporopollens. So that readers can evaluate the significance of the work.

Response to Reviewer Comments

Reviewer #1 (Remarks to the Author):

The authors demonstrated in this manuscript that PVA-based cross-linked polymers were synthesized as novel robust materials inspired by sporopollenin. The synthetic strategy to mimic the plant-derived stiff biopolymer is rationally designed to achieve a biomimetic chemical structure resembling the sporopollenin. The structural analysis on the synthesized polymers seems to scientifically sound well, but I have uncertain points in the material evaluation. The detailed comments are listed below:

We thank Reviewer #1 for their review of the manuscript and supporting information.

1) The plausible chemical structure of sporopollenin, which was revealed by the authors in previous work, contains fatty acid and alpha-pyrone moieties at the terminal of polyketide backbone. According to the chemical composition in the previous work, I think these components cannot be ignored for the design of sporopollenin-mimetic polymers. Why did the authors omit such components for this work?

A number of factors influenced our decision to exclude the proposed fatty acid and pyrone moieties from our synthetic analogues: (1) our primary interest in this work is the exploration of the effects of variation in the crosslinker moiety, (2) synthetic access to the proposed backbone is non-trivial, requiring detailed control of each stereocenter of the skipped polyol backbone, (3) the goal of the work is to produce polymers of potential utility to the field and, as such, use of a backbone that is both unavailable commercially and synthetically challenging to access would greatly limit the accessibility of the work to the broader field.

2) The authors claimed that the combination of ester and acetal linkages in the cross-linked structure of sporopollenin is quite important for the stability of this biomaterial in the previous study. Why not the authors try to incorporate ester linkages (probably based on fatty acid moiety in sporopollenin)?

Although pine sporopollenin features a combination of both ester and acetal linkages as revealed by our prior work (ref 35), the importance of ester bonds to the stability of the biopolymer is unsubstantiated. Averaged over the structure, there are many more acetal C–O than ester linkages providing stability to the polymer, as plainly shown in the prior work. In the practical sense, the nature of the ester linkage remains, as yet, unclear (i.e., to what does the ester link?), making synthetic recapitulation intractable at this stage. Finally, the use of long PVA backbones ostensibly replaces the comparatively weak ester bond with a strong C–C bond. If anything, we expect our synthetic polymers to exhibit greater stability than natural pine sporopollenin.

3) In TGA of the synthetic sporopollenin analogues, the authors concluded that the weight loss at around 200 oC is attributed to residual solvent DMSO. However, the weight loss at 200 oC reaches 35% for all the samples, which means the materials are organogels rather than cross-linked polymers. Is there possibility that additional thermal condensation occurred during TGA process? I recommend the authors to analyze the exact amount of residual solvent by other methods (NMR, elementary analysis, etc). Such the large amount of residual solvent should also affect the mechanical properties of the polymers.

We have clearly noted the % weight loss attributed to DMSO in both the manuscript and SI. We disagree with the reviewer's notion that the state of being an organogel and being crosslinked are mutually exclusive. Crosslinking of our polymers does occur during synthesis, as exclusion of the crosslinker from the synthesis conditions does not result in a solid material. Further, the acid catalyzed condensation of PVA with monoaldehydes is well established (i.e. the synthesis of PVB). While it is possible that additional condensation occurs during TGA, we assert that any number of thermally facilitated reactions may occur during the course of TGA on polymers of nearly any description and, thus, is simply the nature of the analytical technique. Ample characterization of our materials is available in the manuscript and SI including, as suggested, NMR, which can be found in Figure 3B. Certainly residual solvent impacts mechanical properties, which is why we analyzed and reported the solvent content during the course of this work.

4) In Figure 4D, the legends for color bars are missing.

The requested legend has been added.

5) For the mechanical properties (Shore hardness, compression study), a control sample (namely, native sporopollenin) should be required for comparative analysis.

We invested considerable time and effort towards the direct comparison of natural and synthetic sporopollenins, however, we concluded that an accurate and meaningful experimental comparison is currently beyond the field's capabilities due to the supramolecular structure of natural sporopollenin. Natural sporopollenin is typically biosynthesized and deposited to the outer wall of plant pollen grains. Thus, compressive testing of natural sporopollenin provides results that are a composite of the contributions of the molecular structure of sporopollenin, as well as the supramolecular structure, that are not easily deconvoluted. As we are not (yet!) able to prepare synthetic sporopollenins with discrete control over their supramolecular structure, our synthetic samples are essentially sheets of the polymer. Compressive testing of these synthetic samples provides results characteristic of the material without contribution from a porous spheroid supramolecular shape and are, therefore, not an apples-to-apples comparison with the natural samples. Only evaluation of the properties of the powdered samples are directly comparable, as the supramolecular structures are destroyed during the crushing process. Our evaluation of degradation by thioacidolysis (SI pg 10) is representative of one such study on the powered natural and synthetic samples. Should the reviewer have suggestions for the deconvolution of molecular and supramolecular contributions to compressive testing data, we would be enthusiastic to test them.

6) The modulus of cross-linked polymers was strongly related to the molecular weight of inter-crosslinking points. Therefore, generally the more cross-linked the polymer is, the higher moduli of the polymers become. As the cross-linking density increased in Table S5 (polymers 13-18), the observed moduli tend to decrease. The authors need clearer explanation about this.

We respectfully disagree with the reviewer's assessment. As shown in Table S5, the moduli of the aforementioned polymers are almost all within a standard deviation of each other. As a result, we make no claims regarding a relationship between crosslinker density and modulus, instead noting a clearer relationship between crosslinker length and modulus.

7) The synthesized polymer 26 containing coumaric acid moiety is the most similar structure to sporopollenin. Since the other analogues are based on PVA cross-linked aldehyde, which are very common polymeric materials (for example, many reports on PVA cross-linked by

glutaraldehyde have been reported), I recommend the authors to emphasize the specific feature of the synthesized polymer 26 on the discussion for material properties.

We first note that our work differs significantly from the referenced glutaraldehyde work in the following ways: (1) we explore a much larger crosslinker chemical space, (2) we focus on linkers with significantly greater flexibility than the relatively small glutaraldehyde, (3) we identify and solve numerous preparative challenges associated with the implementation of water-insoluble crosslinkers, (4) as the reviewer mentions, we explore substituents on the crosslinker and provide a blueprint for the preparation of myriad crosslinker analogues, and (5) our results provide insight into a poorly understood natural polymer. We believe these components provide the work with ample novelty beyond glutaraldehyde crosslinked PVA. We further note that we have already highlighted the key contribution of the coumaric acid moiety in the manuscript, stating "It is notable that in all instances observed, synthetic analogue **26** demonstrated decreased modulus compared to the analogous simplified synthetic analogue **15**, suggesting that the presence of *p*-coumaryl substituents suppresses inter-/intramolecular interactions of analogues, thereby leading to reduced modulus."

8) Comment: I am just curious about the role of coumaric acid moiety. Cinnamic acid analogues generally dimerize under light irradiation, making robust cross-linking if they are incorporated in polymer structures. Do the authors think that such the dimerization of coumaric acids can take place in the synthesized polymers (and also in native sporopollenin)?

This is an interesting point! Given that our prior work (ref. 35) on the degradative thioacidolysis of sporopollenin revealed no dimerized crosslinkers, we lack evidence of the suggested coumaric acid photodimers. It may be that the coumaryl moieties are, by and large, too far apart for efficient photodimerization in both natural and synthetic materials. Currently, it is hypothesized that the coumaric acid moieties of natural sporopollenin act to absorb UV light that would otherwise crosslink the DNA of the fragile plant gametes contained within.

Reviewer #2 (Remarks to the Author):

Weng et al. describe the bioinspired synthesis of a series of sporopollenin with discrete control over linker length, linker substituent(s), degree of crosslinking, and polyvinyl alcohol backbone properties, including average molecular weight, polydispersity, and tacticity, measured chemical, thermal, and mechanical properties of the synthetic sporopollenins. This may provide chemical tools for the sporopollenin biosynthesis, and could encourage the development and application of robust sporopollenin-inspired polymers. However, the authors should address the following concerns.

We thank Reviewer #2 for their review of the manuscript and supporting information.

1. In Figure 1, author shows electron micrograph detailed hypothesis for the molecular structure of P. rigida sporopollenin. This could perhaps go to supplementary information as the structural model of the sporopollenin have been confirmed by authors' previous article (Nat. Plants 2019, 5, 41.).

Given both the lack of familiarity most readers will have with sporopollenin as a biopolymer and the importance of our group's prior structural hypothesis to the nature of the work disclosed, we would prefer to have the opportunity to introduce the reader to both in the introduction of the

manuscript. We have provided appropriate citation in the figure legend to ensure readers are aware that those particular items represent prior work.

2. The authors have obtained a series of sporopollenin-inspired polymers from chemical synthesis with different linker carbon count, linker density and PVA MW, that is basis for selecting these parameters. Why not we can see the combination of linker carbon count (8 #C), linker density (10 %) and PVA MW (104500 g/mol)?

The compositions for synthesis were selected such that a single variable was modified for each series to permit observation of trends. Thus, three sets were selected: (1) Linker carbon count varied, (2) Linker density varied, and (3) PVA average MW varied. The structural features not being varied were held at values that most closely reflect authentic sporopollenin (in the case of linker density and linker carbon count) or an intermediate value for the series (in the case of PVA average MW). If you prefer to think in terms of chemical space, these series describe three lines, intersecting at 20% linker density, C16 linker carbon count, and 93,500 g/mol PVA average MW, in a 3D space with axes of linker density, linker carbon count, and PVA average MW. That makes 120 possible combinations (5 carbon count options x 6 density options x 4 PVA options), the synthesis of which in totality was not necessary to explore the structural repercussions of individual variabilities. That being said, our work makes abundantly clear that, should a C8, 10%, 104,500 g/mol sample be desired, it can certainly be prepared.

3. Information on the number of tests is missing and needs to be given (number of specimens of the thermal characterization, number of mechanical properties, and number of swelling ratios).

This information is found in the methods section and is also found in the legend of Figure 4. N=3 for all the mentioned analyses. The indication of analysis in triplicate was omitted in the method section for TGA and has been updated accordingly.

4. The uniform hardness of sporopollenin-inspired polymers averaging 71 ± 5 Shore D regardless of crosslinker length, crosslinking density and PVA average molecular weight, what is the hardness of natural sporopollenin?

To the best of our knowledge, this cannot be measured accurately as a result of sporopollenin’s size and supramolecular shape. For a fuller description of the problem, please see our response to comment #8.

5. What are the key factors affecting compression performance of sporopollenin-inspired polymers? There is little difference in compression performance between the various sporopollenin analogues.

As noted in the manuscript, we observed that crosslinker length appeared to have an effect on compressive modulus, with the C12 material providing the highest value. We also note that the presence of the coumaryl group decreases stiffness as compared to the analogous unsubstituted linker. While the differences may not be dramatic, they are notable given the subtlety of the changes explored.

6. The authors have compared relationship between weight swelling ratio and crosslinker density. Are there any relationships between swelling ratio and linker carbon count PVA MW?

No obvious trends were observed when comparing swelling ratio to linker carbon count and PVA average MW. This is consistent with our expectations given prior studies exploring

crosslinker density to swelling ratio in other polymer systems. A sentence has been added to the manuscript to clarify this point.

7. There are a series of three mass losses, so three corresponding to which number of sporopollenin analogue? Is there relationship between weight losses and synthetic?

As shown in the SI (Figures S44–S57), the TGA thermograms for sporopollenin analogues 6–18 and 26 all show three mass losses. We felt it appropriate to summarize, given the similarities. The reasons for each weight loss from the synthetic materials are indicated in the manuscript.

8. It is necessary to compare the performance between natural and synthetic sporopollens. So that readers can evaluate the significance of the work.

We invested considerable time and effort towards the direct comparison of natural and synthetic sporopollenins, however, we concluded that an accurate and meaningful experimental comparison is currently beyond the field's capabilities due to the supramolecular structure of natural sporopollenin. Natural sporopollenin is typically biosynthesized as a porous spheroid, thus, compressive testing of natural sporopollenin provides results that are a composite of the contributions of the molecular structure of sporopollenin, as well as the supramolecular structure, that are not easily deconvoluted. As we are not (yet!) able to prepare synthetic sporopollenins with discrete control over their supramolecular structure, our synthetic samples are essentially sheets of the polymer. Compressive testing of these synthetic samples provide results characteristic of the material without contribution from a porous spheroid supramolecular shape and are, therefore, not an apples-to-apples comparison with the natural samples. Only evaluation of the properties of the powdered samples are directly comparable, as the supramolecular structures are destroyed during the crushing process. Our evaluation of degradation by thioacidolysis (pg 10) is representative of one such study on the powered natural and synthetic samples. Should the reviewer have suggestions for the deconvolution of molecular and supramolecular contributions to compressive testing data, we would be enthusiastic to test them.

REVIEWERS' COMMENTS:

Reviewer #1 (Remarks to the Author):

The authors responded to all my concerns and revised the manuscript. I understood the fully detail of the concept and challenges in material design, although I little bit disappointed that the authors cannot perform the comparative study with a native sporopollenin, which I believe is the necessary point to convince these novel materials as bioinspired one. Judging from the revised manuscript, I think the manuscript is worth of acceptance in this stage (especially in terms of the synthetic part) and I hope the authors try to develop a more complex sporopollenin-like composite material showing excellent properties comparable with native one in near future.

Reviewer #2 (Remarks to the Author):

The revised manuscript has addressed all the reviwers'comments. I recommend the revised manscript for publication.

Response to Reviewer Comments

Your manuscript titled "Sporopollenin-inspired Design and Synthesis of Robust Materials" has now been seen again by two of our original referees, whose comments appear below. In light of their advice I am delighted to say that we are happy, in principle, to publish a suitably revised version in Communications Chemistry under the open access CC BY license (Creative Commons Attribution v4.0 International License).

Thank you.

We therefore invite you to revise your paper one last time to address the remaining concerns of our reviewers. Reviewer #1 still has some concerns about the bioinspired materials, and we feel that they should be carefully discussed in the Introduction part by expanding on the supramolecular feature of natural sporopollenin and focusing more on the synthesis and characterization. In the meanwhile, Discussion part should include some additional discussion about the limitation of this study and potentially further studies that need to be undertaken in the future.

Per Reviewer #1's request, we have added various discussions in the Introduction and the last section of the manuscript regarding the supramolecular feature of natural sporopollenins and the limitation of this study in comparing the physical properties of synthetic sporopollenin analogs to natural sporopollenins.

At the same time we ask that you edit your manuscript to comply with our journal policies and formatting style in order to maximise the accessibility and therefore the impact of your work.

We have edited our manuscript accordingly.

EDITORIAL REQUESTS

* Your manuscript should comply with our policies and format requirements, detailed in our style and formatting guide (https://www.nature.com/documents/commsj-phys-style-formatting-guide-accept.pdf).

Done.

* Please edit your manuscript according to the editorial requests in the attached table, and outline revisions made in the right hand column. If you have any questions or concerns about any of our requests, please do not hesitate to contact me. It is important that each request be addressed in order to avoid delays in accepting your manuscript. Please upload the completed table with your manuscript files.

Done.

* An updated editorial policy checklist that verifies compliance with all required editorial policies must be completed and uploaded with the revised manuscript. All points on the policy checklist must be addressed; if needed, please revise your manuscript in response to these points. Please note that this form is a dynamic 'smart pdf' and must therefore be downloaded and completed in Adobe Reader. Clicking this link will download a zip file containing the pdf.

Done.
